# Current methods in the diagnosis of invasive meningococcal disease

**DOI:** 10.3389/fped.2025.1511086

**Published:** 2025-04-22

**Authors:** Ergin Ciftci, Duygu Ocal, Ayper Somer, Hasan Tezer, Dilek Yilmaz, Sirac Bozkurt, Oldac Uras Dursun, Şeyhmus Merter, Ener Cagri Dinleyici

**Affiliations:** ^1^ Division of Pediatric Infectious Diseases, Department of Pediatrics, Faculty of Medicine, Ankara University, Ankara, Türkiye; ^2^ Department of Medical Microbiology, Faculty of Medicine, Ankara University, Ankara, Türkiye; ^3^ Division of Pediatric Infectious Diseases, Department of Child Health and Diseases, Faculty of Medicine, Istanbul University, Istanbul, Türkiye; ^4^ Department of Pediatric Infectious Diseases, Faculty of Medicine, Gazi University, Ankara, Türkiye; ^5^ Department of Pediatric Infectious Diseases, Izmir Katip Celebi University, Izmir, Türkiye; ^6^ Pfizer Vaccines, Pfizer, Türkiye; ^7^ Department of Pediatrics, Faculty of Medicine, Eskisehir Osmangazi University, Eskisehir, Türkiye

**Keywords:** *Neisseria meningitidis*, invasive meningococcal disease, laboratory tests, diagnostic, culture, PCR, whole genome sequencing

## Abstract

Invasive meningococcal disease (IMD) remains a significant health concern due to its global distribution, potential for epidemic spread, unpredictable nature, rapid progression, and high mortality rates or permanent sequelae. The global elimination of meningococcal illness *via* immunization is a primary objective of the World Health Organization's strategy to defeat meningitis by 2030. Timely recognition of meningococcal infection and immediate, precise, and specific identification of *Neisseria meningitidis* are essential for optimal clinical management and enhanced outcomes, monitoring evolving meningococcal epidemiology, detecting outbreak activity, and providing an effective public health response. Clinical findings, microscopic findings, Gram stains, and cultures are traditional and widely used diagnostic methods for the definition of IMD, despite some disadvantages. Real-time polymerase chain reaction (rt-PCR) and whole genome sequencing (WGS) are more accurate techniques for the identification of *N. meningitidis* and subsequent investigation; however, their cost and limited availability present issues. WGS has numerous uses, including strain characterization, population genomics, antibiotic resistance monitoring, and outbreak investigation. New-generation molecular technologies have been and will be used for designing meningococcal vaccines, as well as to monitor dynamic molecular meningococcal seroepidemiology. Microbiology reference laboratories are important, and the digital records and expertise they provide benefit public health for *N. meningitidis*, as well as other pathogens. While there has been significant progress in the development of meningococcal infection diagnostic tools, it is probable that a combination of approaches or new strategies will still be necessary. The goal of this review was to evaluate the current methods for diagnosing IMD and to discuss diagnostic challenges in practice.

## 1 Introduction

Invasive meningococcal disease (IMD) is a serious infection caused by *Neisseria meningitidis* (1). Although it is relatively rare, IMD remains a significant health concern due to its global distribution, potential for epidemic spread, unpredictable nature, rapid progression, and potentially fatal course despite treatment (1). The majority of meningococcal meningitis and septicemia cases arise in children under the age of five. Adolescents and young adults are another age group in which IMD is common, as well as being the group with the highest carrier rate (2). Older adults are another age group in which IMD is common (accounting for up to 25% of cases). They have the highest case fatality ratios (CFRs) and substantial sequelae in survivors, which is likely because they have more underlying conditions and less typical presentations, making it harder to get them treated quickly (3, 4). Conditions with an increased risk of IMD include functional or anatomic asplenia, human immunodeficiency virus (HIV), complement deficiency, patients who receive complement inhibitors (e.g., eculizumab), military recruits, and microbiologists who work with *N. meningitidis*. Additionally, there has been a rise in the number of cases of IMD taking place during mass gatherings (5, 6). It is hard to obtain accurate numbers on how common IMD is around the world because of limited notifications in many parts of the developing world and differences in bacteriological surveillance between countries (2, 7).

Diagnosing IMD is frequently difficult due to its first clinical presentations resembling less severe conditions (8). Mortality and sequelae rates are high, even if appropriate treatment is provided. However, due to the disease course, timely and precise identification is critical, and laboratory confirmation and characterization of *N. meningitidis* are essential in all age groups due to the unpredictability of the disease at presentation and difficulties in clinical diagnosis (9–11). Additionally, accurate diagnosis of the disease would contribute to accurate surveillance data, which would also guide health authorities in terms of managing epidemics and planning immunization programs (11). The goal of this review was to evaluate the current methods for diagnosing IMD and to discuss diagnostic challenges in practice.

## 2 Neisseria meningitidis

IMD is caused by the *N. meningitidis* encapsulated Gram-negative aerobic diplococcus of the *Neisseriaceae* family (1, 12, 13). The genetic plasticity and phenotypic diversity are defining characteristics of meningococcus evolution (14). The genome of *N. meningitidis* ranges from 2–2.2 Mb and has approximately 2,000 genes (1, 13). Humans are the only natural reservoir for *N. meningitidis* (2, 13, 15). *N. meningitidis* is a commensal organism in the nasopharynx, especially adolescents and young adults, with up to 25% of individuals being asymptomatic nasopharyngeal carriers and the organism unable to survive outside of the human body (13). Meningococcal colonization can last several months, with person-to-person transmission occurring *via* respiratory droplets or secretions (1). Factors contributing to the transition from carriage to meningococcal infection are not well elucidated, although several virulence factors are known to facilitate binding of the host epithelium, microcolony formation, and, ultimately, invasion into the bloodstream and/or through the blood−brain barrier (1).

Although many meningococcal strains are unencapsulated, invasive strains generally express polysaccharide capsules. Twelve antigenically and chemically distinct meningococcal serogroups (A, B, C, E, H, I, K, L, X, W, Y, and Z) are defined based on the structure of the capsular polysaccharide (1). Six serogroups (A, B, C, X, W, and Y) account for the majority of global cases of IMD, albeit with differing geographical prevalence (1, 16, 17). The previously identified serogroup D was later ascertained as an unencapsulated meningococcal serogroup C variant (1). Serogroups E and Z are rarely reported but predominantly occur in immunocompromised patients or carriers (12). Serogroups W and E were amended from their original designations of W-135 and 29E, respectively (1). Some isolates either do not react with any specific antisera or react with multiple antisera, leading to their classification as non-groupable (NG) (9).

Meningococci survive phagocytosis and complement-mediated lysis through their capsules (13). The capsule, a significant virulence component of meningococci, is encoded by the capsule polysaccharide synthesis (cps) island. Type IV pili on the surface of encapsulated meningococci enhance attachment to brain endothelial cells, enabling meningeal invasion (1). *N. meningitidis* naturally generates outer membrane vesicles (OMVs) that encompass nucleic acids, outer membrane proteins, and periplasmic proteins. OMVs facilitate the evasion of the host's immune response (13). The virulence factors of *N. meningitidis* include components crucial for immune evasion (resistance to phagocytosis, complement-mediated lysis, and immune escape); niche competition (nutrient transport systems, iron acquisition systems, polymorphic toxins, outer membrane porins, and efflux pumps); and adhesion (13).

## 3 Meningococcal epidemiology

Meningococcal epidemiology is very dynamic and varies geographically, temporally, and by age and serogroup (18). Serogroup A (MenA) was one of the most frequently detected serogroups in the past, especially in sub-Saharan Africa, but is no longer a major cause of epidemic meningitis after the introduction of the MenA-TT conjugated vaccine (MenAfriVac) in the endemic region. The predominant serogroups currently are primarily MenC, MenW, and MenX, which may be associated with outbreaks in Africa (1, 2, 16, 18). Serogroup B (MenB) was a predominant cause (∼60%) of IMD in many global regions, typically affecting infants and young children (1, 18). During the past 10 years, some countries have introduced MenB vaccines; there are promising results showing a reduced number of cases in the vaccinated cohort (19). Historically, MenC was prevalent in developed countries, sporadically causing outbreaks and epidemics (2). Following the widespread implementation of MenC vaccination programs, its prevalence has significantly declined. However, during the mid-2010s, serogroup C still accounted for more than 20% of global IMD cases (18). In recent years, there has been a significant rise in cases of IMD attributed to serogroup MenW, perhaps encompassing the hypervirulent ST11 complex, which is linked to an unusual presentation and a high CFR (2). Many countries have implemented MenACWY routines or risk-group vaccination programs to address MenW disease increases (1, 16, 20). IMD due to MenY is also increasing and primarily affects the elderly (3, 18). MenX is rarely reported worldwide in parts of Africa as an outbreak strain (16, 21). MenH and MenZ are rarely reported as a cause of IMD, and MenE has been reported as a carrier strain after vaccine implementation in the Netherlands (18, 22). Meningococcal epidemiology remains unpredictable, and *N. meningitidis* has outbreak potential with new or hyperinvasive clones (1). Following the implementation of rigorous COVID-19 infection control and lockdown measures worldwide in 2020, there has been an effect on the occurrence of IMD, its surveillance, and a decline in vaccination uptake. Incidence rates and corresponding mortality decreased across multiple countries in 2020 (23). After easing restrictions, IMD usually came back because of serogroups that were common before the pandemic and mostly affected unvaccinated age groups, especially adolescents and young adults, and exceeded pre-pandemic levels in some countries (18, 23). Recent meningococcal epidemiology has featured additional shifts beyond serogroup-specific trends (1, 24, 25). The primary objective of the World Health Organization's (WHO) strategy to defeat meningitis by 2030 is to achieve a world free of meningitis, with three key goals: eliminating bacterial meningitis epidemics, reducing cases of vaccine-preventable bacterial meningitis by 50% and deaths by 70%, and reducing disability and improving quality of life after meningitis. This endeavor underscores the urgent requirement to enhance meningococcal vaccination initiatives targeting key disease-causing serogroups and age-specific risk populations (1).

## 4 Complications and mortality

Early initiation of effective parenteral antimicrobial therapy, such as ceftriaxone or cefotaxime, should occur until definitive microbiological confirmation is obtained; the need to cover all potential pathogens exists (2). Although *N. meningitidis* is susceptible to antibiotics, reports have recently emerged of strains resistant to penicillin, rifampicin, ciprofloxacin, and cefotaxime (2, 26–28). The possibility of an increase in antibiotic-resistant strains should be considered in the future. Antibiotic use has significantly decreased mortality associated with IMD; however, despite timely and suitable antimicrobial intervention, some patients may experience rapid disease progression, leading to multi-organ failure and death within a few hours (11). Without treatment, the CFR can reach 50% (13). The CFR remains high (8.3%, ranging from 4.1%–20.0%), particularly in patients with meningococcemia, and it also varies depending on the serogroup (MenB 6.9%, MenW 12.8%, MenC 12.0%, and MenY 10.8%), clonal complex (4%–27.8% for W:cc11) (29). The individual's age is associated with varying percentages: 9.0% in infants, decreasing to 7.0% in 7-year-olds, then increasing to 15.0% in young adults aged 28 years. This percentage stabilizes between 15% and 20% in middle-aged adults, ultimately reaching a peak of 32.8% in elderly individuals (3).

Survivors of IMD experience a wide array of sequelae across all age demographics (30). Digital or limb loss due to gangrene, extensive skin scarring, orthopedic abnormalities such as abnormal bone growth, neurosensory hearing loss, mild to moderate cognitive impairments, and severe neurological disorders or seizures are both short- and long-term sequelae associated with IMD (2, 11, 30, 31). Affected children and their families may experience considerable neuropsychological consequences, such as post-traumatic stress disorder, depression, psychosis, diminished educational performance, and significant anxiety (2). IMD results in diminished quality of life for survivors, their families, caregivers, and the broader community, even in the absence of identified sequelae (32).

## 5 Clinical diagnosis

The early diagnosis of IMD presents challenges and necessitates a significant level of clinical suspicion (8). IMD has various clinical presentations, and these presentations vary according to patients' age. The clinical presentation of IMD exhibits a wide spectrum, ranging from mild febrile illness to severe conditions such as meningitis and sepsis (33). A straightforward classification encompasses meningitis, meningococcemia, and a combination of both conditions, with the potential for patient progression from one to another throughout the course of the disease (2). Relatively fewer common manifestations of meningococcal disease include septic arthritis, pneumonia, myocarditis, endocarditis, pericarditis, endophthalmitis, conjunctivitis (especially in the newborn period), epiglottitis, necrotizing fasciitis, or urethritis (1, 2, 8, 10). Different clinical presentations have also been reported according to serogroups. Presentation with meningitis alone is mostly associated with MenB (34). MenW cases present more frequently with septicemia in all age groups (35). MenW and MenY primarily impact older patients, and the infection typically presents as bacteremia or pneumonia (4). The changing epidemiology of IMD can be associated with changing clinical presentations, as certain strains appear to be more commonly linked to atypical clinical manifestations (12).

The incubation period ranges from 1–10 days, with a typical duration of 3–4 days (11). During the first phase (the first 6–9 h), typically considering other common infections, patients may present with nonspecific signs and symptoms such as fever, sore throat, coryzal symptoms, sudden onset myalgia, anorexia, tachypnea, abdominal pain, nausea, vomiting, diarrhea, lethargy, and irritability (2, 8, 11, 36). Signs and symptoms in young children may be more insidious and make diagnosis difficult. At these ages, irritability, lethargy, and poor feeding may be the main findings, and seizures with focal onset and bulging fontanel may be observed (2, 8, 37). The classical signs and symptoms of meningitis, including fever, headache, photophobia, neck stiffness, and altered mental status, are more prevalent in older children and may be incomplete or absent altogether (2). In adolescents, general symptoms of sepsis, such as leg pain, cold extremities, and abnormal skin color, commonly manifest within the initial 12 h (median onset 7–12 h), especially in cases of severe meningococcemia. In contrast, classic features, including hemorrhagic rash, meningism, and impaired consciousness, typically present as later signs (median onset 13–22 h) (38). Findings indicating a high risk for IMD include neck stiffness, a non-blanching rash, leg pain, photophobia, and a low Glasgow Coma Scale (GCS) score (36). Symptoms such as cold extremities, prolonged capillary refill time, pain in the extremities of the upper and lower limbs, reduced urine output, and abnormal skin coloration can be observed (2, 12). The disease progresses more rapidly in such cases, and multiple organ failure and death may occur within 24–48 h after developing symptoms (8).

The presence of a classical hemorrhagic rash in a child presenting with fever strongly indicates the likelihood of IMD (2). It has been reported that hemorrhagic skin lesions are seen in 28%–77% (40%–90%) of patients with IMD. The initial cutaneous manifestations may mimic a viral maculopapular rash, while the distinctive hemorrhagic skin lesions may appear later as the disease progresses. In 20% of patients, these lesions do not manifest. It has also been reported that whether the patient's skin is hyperpigmented may affect the detection of skin lesions (39). Lesions are detected most often on the trunk and extremities but can spread, involving any part of the body. All lesions may progress to larger ecchymosis lesions. In severe cases, lesions may burst and lead to necrosis (8).

Meningitis represents the most prevalent clinical manifestation of IMD, occurring in 30%–60% of cases (2, 40). In the presence of meningitis, symptoms such as headache, decreased GCS, neck stiffness, photophobia, seizures, neurological findings, and a bulging fontanel in infants may occur (36). Children may initially have only fever and vomiting or lack specific manifestations altogether. Only 27% of patients reveal the classical triad of neck stiffness, fever, and altered consciousness. Focal neurological abnormalities occur in as many as 20% of individuals with meningococcal meningitis (41). Patients might show signs of meningeal irritation, potentially advancing to decreased consciousness, coma, seizures, and symptoms of elevated intracranial pressure. Meningitis can arise after an extended duration of low-grade bacteremia and may present with a less severe progression compared to shock (2).

Approximately 30% of cases present with the most severe manifestation of IMD: meningococcemia without meningitis. Sudden onset of fever, non-blanching rash, purpura fulminans, cold extremities, myalgia, leg pain, low GCS, tachycardia, and shock may occur (41). The onset of hypotension indicates a breakdown in compensatory circulatory mechanisms. The disease can quickly advance to cardiovascular failure, disseminated intravascular coagulopathy, acute kidney injury (potentially necessitating renal replacement therapy), adrenal hemorrhage, multi-organ failure, and death, unless prompt and vigorous resuscitation, along with organ support, is started (2). Atypical clinical presentations can result in misdiagnosis, often correlating with elevated CFRs due to delays in optimal management. The onset may manifest as early abdominal symptoms, including abdominal pain, gastroenteritis, diarrhea, and peritonitis (12). This presentation is currently on the rise and appears to correlate with the recent emergence of serogroup W/CC11 isolates across several continents, which are associated with increased CFRs (12).

Meningococcal pneumonia remains poorly understood and significantly underreported. Approximately 5%–15% of all IMD cases are estimated to occur, with a higher prevalence in the elderly compared to children. Meningococcal pneumonia is characterized by fever, dyspnea, cough, radiologic findings, and the identification of *N. meningitidis* in the blood through culture and polymerase chain reaction (PCR), indicating bacteremic pneumonia (12). The positivity rates of blood cultures in diagnosing meningococcal pneumonia vary significantly, ranging from 6%–79.3%. Sputum is an unreliable diagnostic tool, as it is challenging to distinguish between asymptomatic oropharyngeal carriers and pneumonia cases caused by meningococcus (42). Pleurisy may also arise in conjunction with the presence of meningococci in pleural fluid. The case-fatality rate of meningococcal pneumonia is significant at 16%, potentially influenced by co-morbidities prevalent in the elderly population. Risk factors encompass age, pre-existing respiratory conditions, human immunodeficiency virus infection, and recent viral respiratory infections, including influenza. Serogroups linked to meningococcal pneumonia may differ from those observed in typical IMD cases, specifically serogroups Y (CC23) and W (CC11) (12).

Meningococci have the potential of causing various forms of IMD and can also be isolated from other sterile sites, including joint fluid, pericardial fluid, and pleural fluid (12). The prevalence of these forms was 6% in Europe (12). They may occur concurrently and present with or without the other typical characteristics of IMD. Septic arthritis represents an extra meningeal manifestation resulting from the hematogenous dissemination of *N. meningitidis*. The detection of meningococci and associated radiological findings is beneficial in diagnosing arthritis (12). The prevalence of arthritis in IMD ranges from 2%–10%; however, the incidence of meningococcal arthritis may be underestimated (12). Gyamfi-Brobbey et al. (43) conducted an analysis of 162 cases of meningococcal septic arthritis in England and Wales from 2010–2020, which accounted for 2% of all IMD cases. Septic arthritis typically presents as a monoarticular condition, predominantly involving large joints, including the knee, hip, or shoulder. Isolates may belong to various serogroups, with a notable predominance of serogroup W within CC11 (12). Strains from Group B exhibited a two- to sixfold reduction in the likelihood of causing septic arthritis compared to strains from Groups W, C, and Y (43). Pericardial involvement represents an additional extrameningeal manifestation. In an IMD episode, the presence of chest pain, tachycardia, polypnea, tamponade, and paradoxical pulse necessitates an ECG to identify abnormalities such as ST segment elevation, along with the requirement for echocardiography (12). The prevalence of meningococcal urethritis is on the rise. Urethritis, while not an invasive infection, has been associated with instances of IMD in recent case reports. This correlation has been observed following urethritis cases linked to serogroup C isolates of the CC11 clonal complex among men who have sex with men (12).

## 6 Laboratory diagnosis of meningococcal infections

Taking appropriate samples for IMD and rapidly delivering them to the laboratory constitute the first and most important step in laboratory diagnosis (9). It is useful for clinics to prepare their guidelines and flowcharts for the definition of suspected and definite cases, the reporting of cases, and the laboratory steps to be followed. There is also a need for a full blood count, serum biochemistry, acute phase reactants (serum C-reactive protein and serum procalcitonin levels), and coagulation tests. Thrombocytopenia and impaired coagulation tests, such as fibrinogen levels, are critical for people with disseminated intravascular coagulation (41).

Recommendations for laboratory diagnosis and confirmation depend largely on the case definition criteria and vary between countries. There are also differences between probable, suspected, and confirmed case definitions (44). In general, the laboratory diagnosis of IMD depends on the demonstration of N. *meningitidis* in samples from normally sterile sites (e.g., cerebrospinal fluid-CSF, blood, synovial, pleural, peritoneal, pericardial) or specific meningococcal DNA sequences in CSF or blood samples with validated techniques (9, 11, 45, 46). The culture of meningococci from a normally sterile site (e.g., blood, CSF, or skin lesions) has traditionally been viewed as the gold standard for IMD diagnosis and confirmation (1, 11, 44).

### 6.1 Sample collection and transport

The appropriate collection and transportation of clinical specimens is essential for isolation, identification, and characterization. Transporting samples under appropriate conditions, delivering them to the laboratory promptly, and handling them with appropriate safety precautions are crucial (9). To preserve viability, it is ideal to obtain clinical specimens prior to antimicrobial therapy. Based on the clinical presentation of the IMD and the specific test required, various samples may be collected, including CSF, peripheral blood, skin lesions, or, when indicated by symptoms, synovial, pleural, or pericardial fluid, as well as naso/oropharyngeal swabs (44).

### 6.2 Blood culture sampling

Obtaining two sets of blood cultures is the first crucial step in a laboratory diagnosis of IMD and sepsis. To minimize the risk of contamination during blood collection, hand hygiene should be maintained, disposable gloves should be worn, and it is important to comply with sanitation conditions. Current recommendations for routine blood culture sampling advise collecting at least two sets, approximately 30–40 ml, distributed into four blood culture bottles—two for aerobic organisms and two for anaerobic organisms—with the aerobic bottles being filled first. It is often not possible to take this amount of blood samples from children, and 1–3 ml can be considered sufficient. Typically, 1–2 ml of blood sample from a child is mixed with 20 ml of the blood culture broth, while 5–10 ml of adult blood is combined with 50 ml of blood culture broth. Inoculated blood culture bottles must be delivered to a microbiology laboratory without delay. If immediate transport to a microbiology laboratory is not feasible, inoculated blood culture bottles should be placed in an incubator at 35–37°C with approximately 5% CO_2_ (or in a candle jar) until transport can be arranged. Blood cannot be transported prior to being placed in a blood culture bottle due to the absence of anticoagulant in the syringes. Multiple factors influence the sensitivity of blood cultures, including the frequency of collections, the volume per collection, measures taken to counteract the bactericidal effects of blood, and the patient's age (9, 44).

### 6.3 Lumbar puncture and CSF sampling

In suspected bacterial meningitis, CSF provides as the optimal clinical specimen for the isolation, identification, and characterization of etiological agents (9). It is crucial to differentiate meningitis according to the causing pathogen (47). If there are no contraindications, patients with suspected meningitis should undergo a lumbar puncture (LP) as soon as possible, and a CSF sample should be obtained for detailed analysis. Prior to LP, the safety of the procedure should be assessed. A negative CSF test does not rule out IMD, and it has no bearing on the diagnosis of meningococcal septicemia without meningeal invasion.

Contraindications for lumbar puncture include coagulopathy due to anticoagulant therapy, severe thrombocytopenia, or uncorrected bleeding diathesis, as well as local skin infections at the puncture site (48). In cases of shock, the priority should be stabilizing the patient, including addressing airway, breathing, and circulation, and initiating appropriate antibiotic therapy. A lumbar puncture in the presence of shock, increased intracranial pressure, and coagulopathy, is potentially dangerous and unlikely to alter initial management. Additional contraindications encompass focal neurological deficits (excluding cranial nerve palsies), papilledema, persistent or uncontrolled seizures, or a GCS score of 12 or lower. Furthermore, suspicion or presence of cerebral edema or focal space-occupying lesions is noted due to the risk of brain herniation (48–51). Some guidelines include an immunocompromised state as a contraindication (49). In some contraindications, after stabilizing the patient's condition, LP may still be necessary for diagnosis. There are numerous guidelines for imaging principles before LP; usually, LP does not require prior imaging, and clinical features can be useful for predicting patients where an abnormal neuroimaging finding is likely. A non-contrast cranial computerized tomography (CT) prior to LP is recommended when there is suspicion of an intracranial lesion with a mass effect, abnormal intracranial pressure due to elevated CSF pressure, or tonsillar herniation, as indicated by medical history or neurological assessment, and in instances of recent seizures, altered consciousness, or papilledema (50). Notwithstanding worldwide guideline recommendations, substantial disparities in a country's available resources may affect patients' likelihood of receiving cranial CT scans. While it is improbable that LP prior to CT would result in hindbrain herniation, a nationwide cohort analysis indicated that only a small fraction of 73 patients who experienced deterioration after LP before CT had a contraindication for the procedure. Another limitation of CT is the lack of documentation of papilledema, which is a criterion in major guidelines (50). CT before LP, despite its potential to delay definitive diagnosis, prolong the time for antibiotic administration, increase costs, and have minimal clinical utility, should still be performed according to guideline recommendations in selected patients with suspected acute bacterial meningitis.

LP is an invasive procedure that must be conducted solely by skilled professionals in aseptic settings, utilizing the necessary personal protective equipment (sterile gloves, surgical masks, etc.) (9, 52). The patient must remain immobile during the procedure, either seated or lying laterally, with the back arched forward to bring the head close to the knees, facilitating the separation of the lumbar vertebrae (9). LP needles need to be selected according to the patient's age. The preferred entry point for LP is between the L4–L5 vertebral spines (9, 52). If possible, three tubes of CSF samples, each of 1 ml, should be collected, with the first tube for biochemistry, the second tube for microbiology, and the third tube for cytology. If only one tube can be obtained, it should be sent to the laboratory for microbiological examination. For Gram staining and culture, it is preferable to use sediment after centrifugation; however, directly using samples less than 1 ml can reduce sensitivity. CSF is normally clear and colorless, and a turbid, cloudy, or purulent appearance favors bacterial meningitis, including IMD (9). CSF samples should be delivered to the laboratory at room temperature within one hour; if this time is exceeded, they should be transported in trans-isolate (T-I) medium. If there is a delay in transportation and T-I medium is not available, the CSF sample should be stored at 35%–37°C with 5% CO_2_ (or in a candle jar) (9). Once the CSF arrives in the microbiology laboratory, if more than 1 ml of CSF is available, it should be centrifuged at 1,000 × g for 10–15 min. The sediment can then be used for Gram staining and primary plating on chocolate agar plates (CAP) and blood agar plates (BAP), and the supernatant can be used for rapid diagnostic tests (antigen detection by latex agglutination). If approximately 1 ml of CSF is available, it should not be centrifuged; instead, the CSF should be plated directly onto CAP and BAP and used for Gram staining (9).

### 6.4 Other sampling

IMD can present in various forms other than meningitis, such as pneumonia, septic arthritis, and pericarditis. Therefore, samples other than blood and CSF may also have diagnostic value. Meningococcal pneumonia is characterized by specific symptoms and signs, alongside the identification of *N. meningitidis* in the bloodstream through culture and/or PCR methods, indicating bacteremic pneumonia (12). This emphasizes the necessity of conducting blood cultures in the presence of such clinical presentations. Diagnosing meningococcal pneumonia presents challenges, as the isolation of the organism from sputum fails to differentiate between carriers and individuals with pneumonia caused by the organism (8). In cases where the blood culture is absent or negative, various criteria can be employed to confirm meningococcal pneumonia in conjunction with the previously mentioned clinical manifestations. This includes the identification of capsulated meningococci as pure culture in deep respiratory samples, such as broncho-alveolar washes, with a concentration of no less than 10^6^ CFU/ml (12). The detection of meningococci in the blood, in conjunction with clinical manifestations and imaging findings from x-ray or echocardiography, can aid in the diagnosis of arthritis and pericarditis (12). The diagnosis of septic arthritis is established through the detection of bacteria *via* culture and/or PCR in the joint. The diagnosis of pleural or pericardial meningococcal infection is established through the detection of bacteria *via* culture and/or PCR in the sterile fluids (12). A positive throat swab culture may serve as corroborative evidence of IMD in sporadic cases, though it is not definitive (45). In clinically suspicious cases with negative cultures, including close contacts, nasopharyngeal swabs may be employed, as they can detect meningococci in up to 50% of cases (44). The isolation of meningococci from the nasopharynx in a patient is not deemed confirmatory; however, a concordance rate of 97% has been observed between nasopharyngeal and invasive isolates. Throat swabs exhibit reduced sensitivity to the effects of prior antibiotic therapy (44). Saliva collection is straightforward, and its integration with quantitative PCR (qPCR) allows for the consideration of detection in meningococcal carriage studies (53).

### 6.5 Microscopic examination/gram staining

In 96% of patients with bacterial meningitis, CSF examination reveals characteristic abnormalities, including pleocytosis and alteration of CSF glucose and protein levels (48). In bacterial meningitis, the white blood cell (WBC) count (with neutrophil predominance) for CSF has been reported as >10 cells/mm^3^ (9). The normal cytology of CSF in infants is characterized by a white blood cell count of 10–30 WBC/mm³, with approximately 50% being neutrophils (9). However, reports have also documented cases of bacterial meningitis with a normal CSF WBC count (54).

Clinical practice should encourage Gram staining due to its affordability, accessibility, ease of application, and speed. In addition, the sensitivity of Gram staining does not change, even in the case of antibiotic administration (10, 45). Gram staining of the sediment after centrifugation yields better results if you take more than 1 ml of a CSF sample (9). *N. meningitidis* can live inside or outside neutrophils. It shows up as Gram-negative, coffee-bean-shaped diplococci that are red or pink (13, 14). Although the specificity of Gram staining is high, at 95%, it has a largely variable sensitivity of 75%; it varies between 30% and 89%, mainly related to low bacterial load or early antibiotic treatment (44). In IMD with high CSF WBC counts, the number of organisms may be so low as to be undetectable (45). Gram stains should be conducted promptly after sample collection, a requirement that may not be achievable in hospitals without round-the-clock laboratory services (44). Gram staining of skin lesions can identify pathogens in patients with suspected IMD, particularly when a lumbar puncture is contraindicated (48). A skin biopsy is a simple and minimally invasive examination that allows Gram staining, skin culture, and PCR to detect *N. meningitidis* (41). The sensitivity of Gram stains on skin lesion samples is 30%–70%. However, this varies depending on the type of IMD and skin lesion, with the highest sensitivity in meningococcemia with hemorrhagic lesions (8, 9, 45, 55). A study indicated a sensitivity of 56% and a specificity of 100% when cultures and Gram stains were assessed concurrently in the skin biopsies of patients with IMD (56). Gram stains of skin biopsy can remain positive for extended durations (approximately 48 h) following antibiotic administration, attributed to the limited penetrability of antibiotics in poorly perfused lesions (45). However, the sensitivity of the test during this period remains undetermined. A negative result does not rule out IMD.

### 6.6 Biochemical examination

CSF biochemistry also gives an idea about the etiology (bacterial or not) but does not make a definitive diagnosis. An increase in CSF opening pressure (>180 mm H_2_O) and turbid, cloudy, or purulent appearance are in favor of bacterial meningitis (9). CSF glucose levels were evaluated along with simultaneous blood glucose levels. A CSF-to-blood glucose ratio of <0.6 is indicative of bacterial meningitis. A CSF protein level of <0.6 g/L (60 mg/dl) reduces the possibility of bacterial meningitis (48). If antibiotics have not been administered beforehand, CSF lactate levels have high sensitivity and specificity in distinguishing between bacterial and viral meningitis, with a cut-off value of 35 mg/dl (57).

### 6.7 Bacterial culture

The bacterial culture of *N. meningitidis* obtained from blood, CSF, or other typically sterile locations offers definitive proof of IMD (44, 45). Culturing provides isolates for strain differentiation and antibiotic susceptibility testing, as well as further characterization with whole-genome sequencing (WGS), and it supports vaccine formulation (44, 47). Bacterial culturing requires 24–48 h to provide a diagnosis. *N. meningitidis* is a fastidious bacterium, and optimal growth occurs at 35°C–37°C with 5%–10% (v/v) CO_2_. *N. meningitidis* grows on both a BAP and a CAP and grows on trypticase soy agar and Mueller-Hinton agar (9). Selective media, including Modified New York City or Modified Thayer Martin medium, are necessary for culturing from non-sterile sites like the throat (45).

Inoculate 1–5 drops of CSF directly onto both a BAP and a CAP within one hour of collection, using only one drop if the CSF has been centrifuged, provided it can be transported to a microbiology laboratory. Chocolate agar supplemented with vancomycin, colistin, nystatin, and trimethoprim is effective for the primary isolation of *N. meningitidis*. A backup broth, such as brain–heart infusion broth with appropriate supplements, must be inoculated with a portion of the sediment pellet. The CSF sediment must be inoculated onto agar plates and incubated at 35°C–37°C in an atmosphere of approximately 5% CO_2_ for a duration of 18–24 h. The plates and broth must be examined daily for a duration of up to 72 h. Prior to conducting any tests, isolates must be examined for growth purity by assessing colony morphology (9, 44, 45).

The inoculated blood culture bottle was sent to a microbiology laboratory for incubation and subculture. *N. meningitidis* is capable of growth on both BAP and CAP. Blood should be cultured in trypticase soy broth or brain–heart infusion broth, supplemented with growth enhancers. These supplements promote the growth of fastidious organisms. The incorporation of chemical inhibitors, such as 0.025% sodium polyanetholesulfonate, into culture media, along with the dilution of blood samples, neutralizes the blood's inherent bactericidal properties and potential antimicrobial agents (9). Assess the blood culture bottle for turbidity at 14–17 h, followed by daily evaluations for a duration of up to 7 days. Turbidity or lysis of erythrocytes may indicate growth; therefore, subcultures onto primary culture media should be performed promptly. The absence of turbidity does not necessarily indicate that bacterial growth is absent. Due to the fragility of N. meningitidis, S. pneumoniae, and H. influenzae, subcultures must be performed on days 4 and 7, regardless of turbidity presence (9).

The growth and colony morphology on BAP and CAP facilitate the presumptive identification of *N. meningitidis*. Colonies of *N. meningitidis* exhibit a gray, unpigmented appearance on a blood agar plate (BAP), characterized by a round, smooth, moist, glistening, and convex morphology, with well-defined edges. *N. meningitidis* manifests as large, colorless to gray opaque colonies on a chocolate agar plate. Before conducting identification and characterization testing procedures, it is essential to inspect isolates for growth purity (9, 14). The identification of *N. meningitidis* can be achieved through an oxidase test, which indicates that *N. meningitidis* is oxidase positive, and by assessing carbohydrate utilization. Cystine trypticase agar (CTA) methods validate the identification of a strain as *N. meningitidis* through the utilization of carbohydrates, including glucose, maltose, lactose, and sucrose. *Neisseria spp.* generate acid from carbohydrates *via* oxidation rather than fermentation. *N. meningitidis* is capable of oxidizing glucose and maltose, but it does not oxidize lactose or sucrose (45). The CDC has discontinued the recommendation for traditional CTA due to various factors, favoring rapid, nongrowth-dependent tests for carbohydrate production instead (11). Definitive identification involves testing for acid production from glucose and maltose, potentially supplemented by enzyme substrate tests, nitrate reduction tests, superoxol tests, polysaccharide/sucrose production tests, and colistin susceptibility assessments (45). The minimum inhibitory concentrations (MICs) of benzylpenicillin, rifampicin, ciprofloxacin, and cefotaxime are systematically assessed using commercial gradient diffusion strip methodology and documented for all submitted isolates (46). Antimicrobial breakpoints are established by the European Committee on Antimicrobial Susceptibility Testing (46).

CSF culture is the definitive method for diagnosing bacterial meningitis, yielding positive results in 85% of cases. Despite the diagnostic value of CSF culture in meningitis cases, practical reports indicate low culture positivity rates (39). Treatment rapidly reduces this percentage, as the CSF quickly clears viable meningococci. Previous studies suggest that the sterilization of CSF may occur more quickly than previously believed within two hours of the first antibiotic dose (58, 59). A negative CSF culture does not rule out non-meningeal IMD. The sensitivity of blood culture in untreated cases of IMD is reported to be 50% (ranging from 40%–80%), decreasing to 5%–20% or lower following antibiotic treatment (45). The sensitivity of combined Gram staining and culturing of skin lesions is approximately 60%–65%, with increased sensitivity observed in hemorrhagic lesions in patients with meningococcemia (45). Contou et al. (60) showed that skin biopsies with conventional culture and meningococcal PCR had a global sensitivity of 88% in adult patients with IMD with purpura fulminans, even after the initiation of antimicrobial treatment. About 50% of cases of IMD may yield meningococci from throat swabs (postnasal or per nasal), and prior antibiotic therapy lessens its impact. Although the sensitivity of the culture method is low (55%–63%) in meningococcal diseases, its specificity is 100% (61). The positive predictive value of bacterial culture approaches 100% for sterile site specimens, but a negative culture does not rule out IMD, as it depends on how well the specimen was collected, transported, and stored before the culture, as well as the stage of the disease and whether it had been treated with antibiotics before (44, 47).

### 6.8 MALDI-TOF MS

MALDI-TOF MS, a laser-assisted desorption ionization time-of-flight mass spectrometry method, is used to speed up the time it takes to identify bacteria isolated by the culture method. The MALDI-TOF MS method reduces the identification time from hours or days with conventional methods to just a few minutes. MALDI-TOF MS is usually used on colonies grown on agar plates, but it can also be used to identify directly from a positive blood culture bottle, which is very helpful for treating sepsis quickly. MALDI-TOF MS can directly identify microorganisms in CSF, but it requires approximately 10^4^–10^6^ CFU/ml of bacteria (62). Analysis of the commercially available library of mass spectrometry profiles revealed a 67% concordance at the species level between MALDI-TOF MS and WGS characterization (63). MALDI-TOF MS is widely utilized for identifying bacteria in clinical samples; however, it currently lacks the ability to differentiate between *N. meningitidis* and various nonpathogenic *Neisseria species*, including *Neisseria cinerea* and *Neisseria polysaccharea* (64). Reports suggest that updating the database of the MALDI-TOF method is necessary for a more accurate identification of *N. meningitidis* (63). The application of the new enriched reference collection markedly enhanced the identification of *N. meningitidis*, achieving a specificity of 92% (95% CI, 75%–98%), compared to 52% (95% CI, 34%–70%) for the manufacturer's database alone (63). McGalliard et al. (65) recently conducted a proof-of-concept study examining the diagnostic capabilities of Fourier transformed infrared (FT-IR) spectroscopy, comparing it with MALDI-TOF MS in conjunction with WGS. The overall prediction accuracy was 99.6% for FT-IR and 95.8% for MALDI-TOF-MS. The analysis of *N. meningitidis* serogroups demonstrated greater efficacy using FT-IR in comparison to MALDI-TOF-MS (65). Further studies are needed as a potential diagnostic tool for FT-IR.

### 6.9 Antigen and antibody tests

The primary antigen tests currently employed to detect *N. meningitidis* include latex agglutination kits, lateral flow assays, and immunochromatographic dipstick diagnostic tests (66). Antigen testing offers numerous advantages, including expedited results and the capacity for point-of-care administration. While antigen tests exhibit lower sensitivity compared to PCR-based testing, they offer the benefits of ease of administration and rapid results, particularly in resource-constrained settings (66).

Latex agglutination widely confirms *N. meningitidis* infection and provides typing information by detecting soluble bacterial polysaccharides in the CSF (9). Several commercial kits are available for latex agglutination testing, which are used to detect soluble bacterial antigens (capsular polysaccharides). Antigens present in CSF can be detected using latex beads coated with specific antibodies (66). The supernatant of the centrifuged CSF specimen should be tested promptly for optimal results (9). The sensitivity of latex agglutination tests for the direct detection of meningococci in CSF samples can range from 32%–96%, particularly in cases of serogroup B (44, 47). Latex agglutination tests have not been widely used in the diagnosis of IMD.

Rapid diagnostic tests (RDTs) to directly test CSF specimens without the need for prior heat or centrifugation have also been developed. Lateral flow tests comprise premanufactured strips containing dehydrated reagents that are activated upon the introduction of a fluid sample. Various types of lateral flow tests incorporate antibodies as recognition elements (47, 66). RDTs can be produced in large quantities and are relatively inexpensive. RDTs using dipstick diagnostic tests are available, and in-field data appear to be promising. A meta-analysis, which included nine studies with 4,533 CSF samples, evaluated the diagnostic value of antigen tests for *N. meningitidis* identification and found a pooled sensitivity of 91.2% and a pooled specificity of 93.8% (66). Five studies (four prospective, one retrospective) used dipstick RDTs to find serogroups A, A/W, and A/C/W/Y; two studies used a one-step vertical flow method to identify serogroups A/W and X, and there was one prospective study (327 participants aged between 3 months and 86 years) that was performed with an immunochromatographic test, MeningoSpeed (BioSpeedia, France). MeningoSpeed is an immunochromatographic assay capable of detecting meningococcal serogroups from CSF samples (67, 68). It can be maintained at ambient temperature, necessitates a low volume of CSF, is executed in 15 min or less, and is user friendly for bedside applications (67). Haddar et al. (67) evaluated the MeningoSpeed test for *N. meningitidis* A/C/W/X/Y antigens in 560 CSF samples collected from five African countries and France. The sensitivity and specificity were 92.7% and 93.8%, respectively. The grouping results were similar, with a sensitivity of 93.0% for serogroup A, 74.4% for serogroup C, 98.1% for serogroup W, 100% for serogroup X, and 83.3% for serogroup Y (67). However, the MeningoSpeed test panel does not include serogroup B (67). Uadiale et al. (69) evaluated the performance of the Pastorex (Bio-Rad Laboratories Inc., France) meningitis kit for the timely identification of *N. meningitidis* serogroup C in Nigeria; the sensitivity was 80.0%, and the specificity was 94.4%. Antigen tests demonstrate great sensitivity and specificity for diagnosing meningococcal meningitis in CSF specimens and can identify various serogroups, exhibiting enhanced sensitivity. They could function as precise diagnostic tools. Antigen tests are rapid and simple to administer, making them suitable for environments with low resources where culturing and PCR facilities are unavailable. The use of antigen tests alongside PCR may function as a concurrent testing approach to enhance overall diagnostic sensitivity (66).

Serogrouping can also be performed using dot-blotting, in which culture samples dried on nitrocellulose paper are probed with serogroup-specific monoclonal antibodies (1). Beyond serogrouping, antibody-based methods can also characterize meningococcal strains according to antigenic differences in major outer-membrane proteins and LOS. PorB serotyping, PorA serosubtyping, and LOS immunotyping can be performed using dot blotting. Twelve distinct LOS immunotypes (L1−L12) have been identified, with different immunotypes generally associated with one or more specific serogroups. Similarly, PorA serosubtypes are classified based on two major variable regions (VR1 and VR2) and one minor variable region (VR3), whereas PorB serotypes are grouped into two classes (PorB2 and PorB3) defined by size and amino acid sequences. Early meningococcal classification systems were formulated as serogroup: serotype: serosubtype (designated as “P1.VR1 variant, VR2 variant”): immunotypes (e.g., B: 15: P1.7, 16: L3, 7, 9) (1, 9).

Serological tests (e.g., enzyme immunoassay) for antibodies to meningococcal antigens have limited clinical relevance in the acute phase of the disease compared to antigen tests (45). Serological testing utilizing an enzyme immunoassay with outer membrane proteins (OMP) as antigens has been employed for retrospective diagnosis of IMD when cultures are negative due to prior antibiotic administration or when CSF is unavailable for PCR analysis. Assays employing the OMP approach demonstrate high accuracy in detecting IMD in adults and older children (aged four years or older) between 5 and 21 days post-onset of initial symptoms. Nonetheless, the assay exhibits somewhat reduced sensitivity in children under four years of age. The OMP assay exhibits a specificity of around 94% and possesses a high negative predictive value, allowing for the exclusion of IMD in clinically indicated but unverified cases (45).

## 7 Molecular methods for the detection of *Neisseria meningitidis*

The limitation of culture-based diagnosis has led to a preference for molecular tests that yield faster results and do not necessitate the presence of live bacteria and target bacterial DNA (9, 14, 47). Molecular tools such as PCR, real-time PCR (rtPCR), qualitative or qPCR, and loop-mediated isothermal amplification assays (LAMP) can address numerous limitations associated with culture-based methods (47). Molecular tools have become the preferred methods in numerous laboratories, enhancing public health measures through standardized laboratory techniques that facilitate the rapid detection of multiple pathogens (47). In the initial decade of the 21st century, there was a shift in focus regarding microbiological typing methods, as phenotypic and serological approaches were progressively supplanted by molecular techniques that catalog genotypes. Multi-locus sequence typing (MLST) and WGS have established themselves as the gold standard for the characterization of *N. meningitidis* isolates and epidemiological surveillance, significantly contributing to the understanding of the population biology of these microorganisms. The advancement of techniques for comprehensive molecular characterization of meningococci in both culture- and non-culture-confirmed cases has equipped researchers with essential tools for improved surveillance, outbreak detection, and effective management of significant disease prevention strategies (70). MLST and WGS are utilized to classify the organism into clonal complexes. New diagnostic modalities for the detection of *N. meningitidis* have emerged. Significant challenges persist in the implementation of these assays, attributed to the variability in laboratory capabilities, a lack of trained personnel, and difficulties in acquiring reagents and equipment, particularly in low-resource countries (47).

### 7.1 PCR for the detection and characterization of *N. meningitidis*

PCR-based methods are widely used for diagnosing and monitoring bacterial pathogens due to their increased sensitivity, specificity, and high-throughput capabilities relative to traditional laboratory techniques (71). Globally, the prevailing trend is to incorporate PCR as a confirmatory method in the case definitions of IMD (44). The use of PCR to identify bacterial DNA (not needing live or intact cells) in CSF and blood has typically become a quick and highly specific alternative to microscopy, Gram stain, and culture, and it often leads to better results when used for confirmation (48). PCR assays have been validated for various sample types, including whole blood (EDTA), coagulated whole blood, CSF, serum, plasma, and joint fluids (46). Uncentrifuged or resuspended CSF deposits are preferred to increase the sensitivity of detection. PCR assays can enhance the laboratory diagnosis of IMD cases by over 30%, and meningococcal DNA in CSF samples has been identified up to 72 h following the initiation of antimicrobial treatment (46). PCR tests for bacterial meningitis are indeed less affected by prior antibiotic use compared to CSF culture tests (44, 46). PCR is also increasingly used for serogrouping and multilocus sequence typing. The Global Meningococcal Initiative (GMI) indicated that both regional/provincial and centralized reference facilities may be appropriate for the implementation of PCR, contingent upon the timely availability of results (i.e., within hours), without replacing culture or alternative methods (44).

Two target genes have mainly been used in both real-time and conventional PCR to detect *N. meningitidis*: the *ctr*A and *sod*C genes (9). The *ctr*A gene (capsule) is highly conserved among the isolates responsible for IMD. The sensitivity of *ctr*A for detecting *N. meningitidis* was 71.6%, and specificity data were not available. However, false positive results may happen because the *ctr*A gene only finds encapsulated meningococci. Given that at least 16% of meningococci lack *ctrA*, a real-time PCR assay was developed and validated to detect all meningococci, irrespective of encapsulation status (9). This assay specifically targets the Cu, Zn superoxide dismutase gene, sodC, which is not genetically linked to the capsule locus. The *sod*C assay can find meningococci that are encapsulated, and it can also find meningococci that are not groupable and do not have an intact *ctr*A, which is what is found in carriage studies. Sensitivity is 99.6/94.7%, and specificity is 100/77.9%. *sod*C is less sensitive in sterile body fluids; it should be used in conjunction with *ctr*A to improve sensitivity (47). There are homologues of sodC in bacteria other than meningococci, such as *H. influenzae* (47). Sirluck-Scroeder et al. (64) highlighted the potential limitations for sensitivity of *ctr*A in the identification of all invasive *N. meningitidis* isolates. Capsule-null (*cnl*) strains lacking *ctr*A might be responsible for IMD, sometimes with the worst outcome. Evidence exists for capsule-independent virulence determinants that may be horizontally transferred to acapsular strains. Sirluck-Scroeder et al. (64) enhanced *ctrA*-targeted PCR by incorporating three additional noncapsular gene targets: superoxide dismutase *sodC*, the phenol metabolism gene *metA*, and the sulfite exporter *tauE*. Among the samples tested, PCR targeting *ctrA* identified 23 out of 32, whereas PCR targeting *metA, sodC*, and *tauE* successfully detected nine samples that were negative for ctrA. The PCR for *tauE* and *sodC* did not detect two samples, while *metA* successfully identified all 32 samples. Additional validation of PCR targeting *metA*, *sodC*, and *tauE* demonstrated 100% specificity when evaluated against various isolates and samples (64). Diene et al. (72) identified 11 genes that are specific to the *N. meningitidis* genome and are present in at least 177 (97%) of the 183 genomes analyzed. Three genes (*metA, tauE*, and *shlA*) were selected for the development of new qRT-PCR assays aimed at detecting *N. meningitidis*. The three qRT-PCR assays demonstrated high sensitivity and specificity, along with good reproducibility when evaluated using plasmid positive controls and genomic DNA extracted from *N. meningitidis* strains. The *metA* and *tauE* qRT-PCRs demonstrated clinical sensitivity and specificity of 100%, as determined through the analysis of CSF samples positive for *N. meningitidis* or other clinically significant bacteria. Although shlA qRT-PCR demonstrated 100% specificity, its sensitivity was limited to 70% (72). Researchers have developed tests using the outer membrane protein gene porA and the capsule null locus (*cnl)* to identify non-encapsulated meningococci, which rarely cause IMD but are typically present in asymptomatic carriers (47). *crgA* is a member of the transcriptional regulator of the LysR family and can be found in *N. gonorrhoeae*. Sensitivity was 93.7%, and specificity was 96% (47). PorA can be found in *N. gonorrhoeae* and may be absent in some meningococci; sensitivity is 96.1%, and specificity is 91.6% (47). No international consensus exists regarding their use, although PCR methods incorporating multiple targets have been developed (44). The disadvantages of PCR are that WGS is not always possible to perform on PCR-positive clinical specimens where culture has not been performed, and it also cannot be used to determine antimicrobial resistance. However, some new techniques/methods are available to do this in limited circumstances where sufficient quantities of DNA are present.

rt-PCR is more sensitive than traditional PCR. It is also easier to use, reduces the risk of lab contamination, gives accurate results in a few hours, and can specifically find meningococcal DNA (73). rt-PCR tests have been approved by the CDC to find *N. meningitidis* and six common serogroups (A, B, C, W, X, and Y). The sensitivity and specificity for diagnosing meningococcal meningitis using a CSF sample range from 89%–100% and 95%–100%, respectively (73). In a study evaluating Gram staining, culturing, and PCR results in CSF samples for the diagnosis of meningococcal meningitis, the sensitivity was 55% for culture and 97% for PCR. It has been reported that the specificity of PCR is 99.6%, and results are obtained within two hours after the start of the test (74). For the diagnosis of IMD, rt-PCR is better than culturing for laboratory diagnosis by at least three times (3.1 times for CSF samples and 3.5 times for blood samples) (75). Another study reported 96% and 100% sensitivity and specificity of rt-PCR in blood, CSF, and synovial fluid samples from patients diagnosed with IMD (73). In a study evaluating the duplex rt-PCR test for the detection of *N. meningitidis* in CSF samples, the sensitivity and specificity were found to be 100%, and it was reported that when rt-PCR was also performed in addition to the standard microbiological methods, the percentage of cases correctly diagnosed with meningococcal meningitis increased to 50.7% (76). The PCR method offers the advantage of simultaneous testing for *N. meningitidis, Streptococcus pneumoniae*, and *Haemophilus influenzae*, with results obtained more rapidly than through culturing (77). The diagnostic accuracy of PCR in CSF was found to be nearly 95%–100% in the case of culture-positive bacterial meningitis due to these three pathogens (48). Unlike PCR assays, which can find a single pathogen, multiplex PCR assays, which can test multiple targets at the same time, like rtPCR assays, have mostly replaced them because they save time and money on supplies (47). rt-PCR is an important tool for antibiotic resistance for *N. meningitidis*. Antibiotic susceptibility analysis, such as through PCR or WGS, may be conducted for penA (penicillin G), rpoB (rifampicin), and gyrA (ciprofloxacin) (44). Pfeifer et al. (78) validated a rt-PCR assay for the rapid detection of the *bla*_ROB_ gene, which is the only widely found β-lactamase and is increasingly prevalent, to predict drug resistance in *N. meningitidis*. This assay offers a specific, sensitive, timely and precise method for detecting penicillin susceptibility in *N. meningitidis*, complementing culture-based approaches.

Recent advancements in multiplex PCR point-of-care panels enable rapid diagnosis within hours. Commercial kits use the multiplex assay method to investigate multiple pathogens simultaneously directly in CSF or blood samples (48). Trujillo-Gomez et al. (79) conducted a meta-analysis on diagnostic test accuracy, encompassing 19 studies with a total of 11,351 participants. The combined sensitivity exceeded 89%, while the specificity surpassed 97% in comparison to two distinct reference tests. Obaro et al. (80) compared the yield from standard bacterial culturing with the BioFire® FilmArray® meningitis or encephalitis (ME) panel for 400 samples of cases (32 specimens were culture positive, six *N. meningitidis*) with suspected acute bacterial meningitis in children aged less than five years. The BioFire® FilmArray® ME panel detected at least one bacterial pathogen in 24.5% of the samples, including *N. meningitidis.* All culture-positive specimens, including *N. meningitidis*, tested positive on the panel. The BioFire® FilmArray® ME panel was more sensitive and rapid than culturing for detecting bacterial pathogens in CSF (80). Myint et al. (81) evaluated the BioFire® FilmArray® ME panel performance using 7,551 retrospective results of patients from 2016–2022. The positive predictive value varied by organism, with the highest being *N. meningitidis.* This study highlights the utility of ME panels in rapid diagnosis, including *N. meningitidis*, particularly in patients pretreated with antibiotics (81). Sundelin et al. (82) conducted an evaluation of the QIAstat-Dx Meningitis/Encephalitis multiplex PCR panel, comparing its performance to that of the BioFire FilmArray panel. The QIAstat-Dx ME Panel demonstrated a positive percent agreement of 100% for *N. meningitidis* in clinical samples. Negative percent agreement values exceeded 99.0% (82). Cuesta et al. (83) also evaluated the QIAstat-Dx ME panel, and the sensitivity and specificity were 96.43% and 95.24% (75.2%–99.7%), respectively. Wagner et al. (84) tested 220 CSF samples using multiplex LightMix rt-PCR to look for *S. pneumoniae, H. influenzae, N. meningitidis, S. agalactiae*, and *Listeria monocytogenes*. A high level of agreement in bacterial identification between culture and multiplex rt-PCR was observed, at 99% (84). The capacity of these tests to concurrently screen for multiple pathogens represents a significant advantage. Current knowledge must be utilized to enhance hospital protocols and logistics, with the objective of initiating treatment within a maximum of one hour of patient presentation (48). These panels are costly, and the majority of tests do not identify serotype or serogroup. The effects of implementing panels in clinical practice require investigation through randomized clinical studies prior to their introduction into routine practice (48).

Kwambana-Adams et al. (85) evaluated raw genomic DNA extracted from dried blood spots and dried CSF spots, which inoculate and dry small volumes of blood and CSF onto filter paper, for the diagnosis of IMD with a rt-PCR. The overall agreement between the analysis of liquid and dried samples was found to be 94.2% for blood and 96.4% for CSF. The study's findings suggest that dried spot samples, which do not need to be kept cold, could be used instead of liquid samples for diagnosing IMD in places with limited resources (85). Dried spot assays may enhance the diagnostic yield of confirmed IMD in Africa and minimize the necessity for cold chains or substantial volumes of clinical specimens (85).

### 7.2 High-resolution melting (HRM)

HRM analysis is a novel post-PCR technique employed to detect variations in nucleic acid sequences. The literature indicates that HRM offers several advantages compared to other genotyping methods, including cost-effectiveness, high sensitivity, and specificity. This method eliminates the need for post-PCR sample processing and maintains a low-cost relative to other sequencing techniques, making it an effective strategy for diagnostic applications in public laboratories addressing acute invasive bacterial diseases, including IMD. The method detects single nucleotide polymorphisms (SNPs) by identifying minor variations in PCR melting curves (86). De Filipis et al. (71) demonstrated that the use of qPCR-HRM for the simultaneous detection of *N. meningitidis, S. pneumoniae*, and *H. influenzae* in clinical samples exhibited comparable sensitivity and specificity. De Souza Santos et al. (86) concluded that qPCR-HRM can effectively differentiate clonal complexes cc103, cc11, cc32, and cc41/44 of *N. meningitidis*. All clinical samples that tested positive *via* conventional PCR were confirmed with 100% accuracy by qPCR-HRM. Of the negative samples, 31% yielded positive results *via* qPCR-HRM (86). This method, when implemented in facilities with existing qPCR equipment, may offer significant advantages, particularly in public laboratories. Reducing the number of samples for sequencing will decrease costs and, importantly, the time needed to identify known or novel cc, thereby enhancing the efficacy of epidemiological monitoring of IMD (86).

### 7.3 Loop-mediated isothermal amplification (LAMP)

LAMP is a form of nucleic acid amplification that utilizes specific looped primers and strand-displacing DNA polymerase (87). It is a simple, fast (under an hour), sensitive, working-in-isothermal-conditions, and inexpensive technique that does not require a thermocycler. LAMP testing enables the detection of fewer than 10 copies of bacterial DNA. LAMP demonstrates greater tolerance to biological fluids than PCR, enabling direct testing of clinical materials. For these reasons, it has been reported that LAMP is a diagnostic method that can be applied in developing countries with limited resources to rapidly rule in and out IMD. The primers for the LAMP assay targeted the *ctrA* region. Simultaneous investigation of six meningococcal serotypes (A, B, C, W, X, and Y) is feasible. Waterfield et al. (87) conducted a systematic review to assess the diagnostic accuracy of LAMP for predicting and diagnosing IMD, which is defined as the identification of *N. meningitidis* from a sterile site, blood, or CSF. This review analyzed 2,243 tests performed on 1,989 children using either rtPCR or bacterial culture. A pooled analysis showed that the calculated sensitivity of the LAMP method was 94%, and the specificity was 100% (87). The test was found to be very accurate (sensitivity = 84%–100% and specificity = 94%–100%) in all studies, no matter what kind of sample was used (87). Fan et al. (88) demonstrated that a pooled analysis of studies utilizing LAMP exhibited high sensitivity (94%) and specificity (100%) for the detection of meningococcus across all studies, with no evidence of publication bias. The sensitivity and specificity of PCR were greater for CSF samples compared to blood samples or oro-nasopharyngeal swabs, whereas the sensitivity and specificity of LAMP remained consistent across all sample types. The sensitivity and accuracy of LAMP techniques may be superior to or comparable with the established reference standards of qPCR and bacterial culture methods. This less expensive and easier-to-use performance would be a chance to detect *N. meningitidis* in developing countries and low-resource areas (87, 88).

Higgins et al. (89) used Tth endonuclease IV along with a unique LAMP primer/probe to create an innovative real-time multiplex LAMP technology known as TEC-LAMP. The assay focuses on the genes that encode a putative cytolysin secretion ABC transporter protein in *N. meningitidis* (NMO_1242; hylB). This assay achieved simultaneous detection of *N. meningitidis, S. pneumoniae, and H. influenzae*, and may facilitate the identification of allele-specific variations both between and within bacterial species in a single test. The TEC-LAMP assay demonstrated complete specificity, with limits of detection for *S. pneumoniae, N. meningitidis, and H. influenzae* recorded at 39.5, 17.3, and 25.9 genome copies per reaction, respectively. The sensitivity for *N. meningitidis* was 100% when compared to single-plex real-time PCR (89). The eazyplex® CSF direct panel, a commercial LAMP assay, is capable of detecting multiple pathogens linked to meningitis, such as *E. coli, H. influenzae,* L. *monocytogenes, N. meningitidis, S. agalactiae*, and *S. pneumoniae*. It is user-friendly, demonstrating a sensitivity of 90.9% and a specificity of 100% (47).

Tavakoli et al. (90) introduced a novel, efficient, and cost-effective method for the quantitative detection of *N. meningitidis*. This approach utilizes a microfluidic, fully paper-based analytical device (μFPAD) that integrates LAMP with ssDNA-functionalized graphene oxide nano-biosensors. The results can be obtained within one hour, with a detection limit of six DNA copies per detection zone. This device provides versatile functions, including qualitative diagnostic analysis, confirmatory testing, and quantitative analysis. The features of the presented μFPAD enable a straightforward, highly sensitive, and specific diagnosis of *N. meningitidis*. This microfluidic method demonstrates significant potential for the swift identification of diverse pathogens in resource-limited environments (90).

Trung et al. (91) recently developed a CRISPR-Cas12a mixture aimed at reducing false positives in the LAMP-based diagnosis of *N. meningitidis*. Under appropriate biochemical conditions, CRISPR-Cas12a and LAMP can function concurrently to precisely detect *N. meningitidis* genetic material at a sensitivity of 40 copies per reaction within a timeframe of less than two hours. Under optimized conditions, the CRISPR-Cas12a system reduces false-positive results, thereby improving the specificity of LAMP assays (91). Huyen et al. (92) assessed the LAMP/Cas12a combination assay using 139 samples obtained from patients suspected of having meningococcal disease. The LAMP/Cas12a combination demonstrated a sensitivity of 91% and a specificity of 99% in 139 samples from suspected patients. The assay demonstrated no cross-reactivity, exhibited specificity for *N. meningitidis*, and successfully detected MenB, MenC, and MenW. Huyen et al. (92) sought to integrate the sensitivity of LAMP with the specificity of CRISPR/Cas12a cleavage to establish a reliable diagnostic assay for the rapid detection of 139 *N. meningitidis* samples. The sensitivity, specificity, positive predictive value, and negative predictive value of LAMP-CRISPR/Cas were compared with real-time PCR assays. Six specific primers for the LAMP assay were developed to target the *ctrA* gene of *N. meningitidis*, which is conserved across all meningococcal serogroups. The LAMP primers exhibited 100% specificity, as they did not amplify DNA from the other bacterial DNA tested. The application of 0.4 M betaine enhanced both the sensitivity and stability of the reaction. LAMP-CRISPR/Cas identified meningococcal serogroups B, C, and W. The assay demonstrated no cross-reactivity and exhibited specificity for *N. meningitidis*. The analysis of 139 samples from suspected patients revealed a sensitivity of 91% and a specificity of 99% for the test. This optimized method can enhance the existing gold standard for the prompt diagnosis of meningococcal meningitis and meningococcemia (92).

### 7.4 Multi-locus sequence typing (MLST)

Multilocus sequence typing (MLST) is the predominant method employed for the molecular characterization of *N. meningitidis* strains and for tracking the dissemination of hypervirulent strains across various nations (86). Genetic sequencing for MLST originally used the Sanger method, which is relatively low throughput and thus suitable for gene fragments. MLST, which characterizes short fragments of various microbial genes, has been used in numerous laboratories for effective isolate characterization. In global surveillance, DNA sequencing methodologies facilitate the molecular determination of antimicrobial sensitivity by identifying allelic variants of specific genes and their corresponding antibiotic resistance or sensitivity (44). This method was initially used to analyze 450–500-bp nucleotide sequences from internal fragments of seven “housekeeping” genes (*abcZ, adk, aroE, fumC, gdh, pdhC, and pgm*), which encode enzymes involved in bacterial metabolism (12, 93). The resulting sequence types (ST) are categorized into clonal complexes (CC) based on the degree of similarity in their allelic combinations. CCs were first defined based on multilocus enzyme electrophoresis and confirmed by MLST (12). Certain STs exhibit a high frequency that is temporally and geographically stable, with STs grouped into clonal complexes based on matching these “central genotypes” at ≥4 of the 7 MLST loci. Distinct alleles at each locus are designated numerical values to generate seven-digit allelic profiles. The detection of newly emergent variants using molecular typing systems is crucial for public health, as it facilitates the monitoring of new clonal complexes (CCs). This is especially significant when these variants exhibit distinct epidemiological or disease characteristics, such as increased CFRs or enhanced transmissibility, or possess antigens that may evade current vaccination strategies (93). MLST is a resource-intensive method, requiring significant investment in equipment and reagents, thereby limiting its application to reference centers and select research laboratories (86).

Core genome multilocus sequence typing (cgMLST) is frequently used to categorize bacterial strains into distinct types for taxonomic and epidemiological purposes, including *N. meningitidis* (94). cgMLSTs, derived from the 1,422 and 1,649 conserved genes in each species, effectively capture the majority of genetic variations within the core genome, rendering them appropriate for analyses ranging from disease outbreak surveillance to species assignments. cgMLST schemes necessitate centralized databases for the nomenclature of novel alleles and sequence types, which must be globally synchronized and entail escalating computational and storage requirements. Zhong et al. presented a distributed cgMLST (dcgMLST) scheme that eliminates the need for a central database of allelic sequences, utilizing it to investigate the evolutionary patterns of both epidemic and endemic strains within the genus Neisseria. They categorized 69,994 Neisseria strains globally into multi-tiered clusters that designate species, lineages, and localized disease outbreaks. *N. meningitidis* is categorized into 168 endemic lineages and three epidemic lineages, which have been responsible for a minimum of nine epidemics over the past century. The analysis indicates that epidemic and endemic lineages have undergone markedly distinct population dynamics over the last century. Epidemic lineages consistently arose from endemic lineages, spread globally, and seemingly vanished swiftly thereafter. The authors proposed a stepwise model for the evolutionary trajectory of epidemic lineages in Neisseria, anticipating that the establishment of analogous dcgMLST schemes would enhance epidemiological studies of other bacterial pathogens (94).

### 7.5 Whole genome sequencing (WGS)

WGS is a significant technique in infectious disease epidemiology, transforming the surveillance of infectious diseases by enabling the tracking and monitoring of pathogen spread and evolution (95). WGS has become an important tool for global meningococcal surveillance in recent years. It helps to find and describe new pathogens, investigate how pathogens spread during outbreaks, and check the expression of vaccine antigens (96). rt-PCR rapidly identifies the causative agent of meningitis; however, WGS allows for a more thorough characterization of the isolate, potentially facilitating an in-depth exploration of the genome of the highly variable species *N. meningitidis* (96). The WGS method provides full-length and high-confidence genome sequences of genes that influence many phenotypic characteristics of isolates, such as serogroups, antibiotic susceptibility, and vaccine antigen expression (97). Using the WGS method in practical application, detection of antibiotic resistance in meningococcal isolates, investigation of the transmission route, characterization of carrier isolates in a population for the purpose of determining the vaccine strategy, characterization of the capsule polysaccharide synthesis locus to determine serogroups, determination of the origin of the agent in epidemics, and utilization in outbreak or cluster scenarios to support public health decisions are possible (1, 27, 93, 96–98). Sotheran et al. (99) demonstrated that WGS effectively characterized IMD isolates, cataloged their genetic variability, enhanced resolution within clonal complexes (CC), and clarified the evolutionary trajectory of CC11. The increased availability and affordability of WGS has initiated a new era in addressing various issues related to the epidemiology of IMD associated with the evolution of this pathogen (96). WGS provides enhanced resolution for the analysis and monitoring of the dissemination of meningococcal genetic lineages (12). WGS facilitated the identification of a newly emerging serogroup associated with IMD, specifically the W/CC11 strain. WGS has effectively differentiated a specific variant of serogroup C/CC11 responsible for an outbreak among men who have sex with men (12).

In the course of WGS analysis, the accurate identification of *N. meningitidis* was confirmed, and serogroups were established using standard serological techniques, subsequently validated by the rt-PCR method (100). Following DNA extraction and isolation, WGS was conducted on the sequencing platform, yielding overlapping sequences of approximately 300 base pairs in length. The genomes of individual isolates were submitted to the PubMLST database and characterized by the BIGSdb platform, focusing on finetyping loci (*porA, fetA*), MLST genes (*abcZ, adk, aroE, fumC, gdh, pdhC, and pgm*), ribosomal protein genes (*rpsA–rpsU, rplA, rplF, rplI–rplX, rpmA–rpmJ*), and MenB vaccine antigen genes (*nhba, nadA, and fHbp*). The isolates were classified into a sequence type (ST), clonal complex (cc), and ribosomal sequence type (rST) based on the allelic profiles of MLST genes and ribosomal protein genes. New gene and peptide variants were manually scanned, followed by curator approval and annotation, before being incorporated into the PubMLST database (100). Genomes were analyzed utilizing the BIGSdb Genome Comparator tool, employing the core genome cgMLST scheme version 1.0 for *N. meningitidis* (100). The distance matrices, derived from the quantity and allelic variability of genes in individual schemes, were generated automatically. Phylogenetic networks constructed using MATS, gMATS, MenDeVAR, MEASURE, and BATS methods facilitate the identification of peptide variants and antigens present in meningococcal vaccines, aiding in the assessment of potential vaccine coverage.

WGS also aims to develop protein-based meningococcal vaccines (96). The first complete whole genome of *N. meningitidis* facilitated the identification of novel protein vaccine candidates through an approach known as “reverse vaccinology.” The new targets, which comprise the neisserial adhesin NadA, the neisserial heparin-binding protein, and factor H-binding protein (fHbp), were integrated with the OMV derived from a New Zealand vaccine strain to formulate the 4CMen B vaccine. Considering the established sequence diversity of fHbp, Trumenba, another protein-based MenB vaccine, was developed to address the two subfamilies of fHbp. WGS is instrumental in assessing the evolving prevalence of vaccine antigens post-vaccine introduction and in detecting escape mutants (96).

Marjuki et al. (98) used a WGS approach to analyze the capsule polysaccharide synthesis (cps) locus and categorize *N. meningitidis* serogroups. The results of WGS and serogroup-specific assays (SASG) demonstrated an agreement of 91%–100% across all serogroups, whereas the agreement between WGS and rt-PCR was found to be 99%–100%. Of the 453 isolates identified as NG through WGS, there was complete agreement among all three methods for those lacking a capsule polymerase gene, with a total of 31 isolates fitting this criterion. This WGS-based serogrouping method offers a thorough characterization of the *N. meningitidis* capsule (98).

The rapid and cost-effective analysis of WGS data provide significant opportunities for improved molecular surveillance, positioning WGS as a standard method for epidemiological investigations of extensive meningococcal strain collections. Genome libraries facilitate pathogen tracking and the evaluation of virulence and antimicrobial resistance. They are particularly useful for documenting potential vaccine coverage and illustrating the effects of noncapsular vaccines (96). WGS is difficult to implement in resource-limited regions because it requires expensive laboratory infrastructure and technical knowledge (101). As sequencing technology improves and becomes cheaper, WGS is likely to become more widely used and replace many tests. This data source, while currently underutilized, is beginning to yield new insights into the genomic plasticity of this human commensal, which may exhibit occasional pathogenic potential. The recent availability of WGS for extensive collections of *N. meningitidis* isolates from diverse sources, along with databases designed for the storage and sharing of WGS data, and the simultaneous advancement of effective bioinformatics tools, have significantly enhanced our understanding of the species' diversity, evolution, population structure, and the emergence of virulent traits. The implementation of WGS is contributing to improved epidemiological surveillance and is crucial for determining the effectiveness of vaccination strategies (96). Achieving the implementation of WGS in LMICs necessitates addressing various technical, financial, and infrastructural challenges. Until that time, it is improbable that laboratories in low- and middle-income countries will routinely perform whole genome sequencing for meningitis diagnosis (47).

### 7.6 Serogroup, serotype, genotype definition

Cultured isolates require systematic serogrouping. The capacity of reference laboratories to serogroup *N. meningitidis* is crucial, as it allows public health authorities to identify manageable outbreaks through vaccination campaigns, ascertain the serogroups responsible for sporadic cases, and monitor the emergence of new serogroups (44). The serogroup is typically determined by slide agglutination serogrouping and rt-PCR (98). Dot blot ELISA using monoclonal antibodies is an option for the identification of capsular polysaccharide antigens by serological reactions (46). Serogroup-specific antisera can identify and group *N. meningitidis* grown in culture. Rarely, some isolates either do not react with any specific antisera or react with multiple antisera, leading to their classification as non-groupable (9). Testing for all available serogroups of antisera in a laboratory is not always practical. Algorithms for testing can be established in laboratories with prior knowledge of the prevalence or absence of serogroups in a specific geographic area, allowing for initial testing of the most common serogroups (9).

The gene *sia*, responsible for sialic acid biosynthesis and also referred to as syn for capsule biosynthesis, is utilized for genotyping serogroups B (*synD*), C (*synE*), Y (*synF*), and W (*synG*). The *sacB* gene is associated with serogroup A, while the xcbA gene, likely encoding the capsule polymerase, is associated with serogroup X. Various systems have been established for the nomenclature of genes involved in meningococcal capsule biosynthesis. Various groups have designated this operon as syn for capsule biosynthesis, while others have adopted the sia nomenclature for sialic acid biosynthesis (9). Additionally, some have referred to these genes as neu genes, drawing on homologies to E. coli K1 genes involved in N-acetylneuraminic acid biosynthesis. Initial assumptions regarding the siaD genes of serogroups B, C, W, and Y as alleles were refuted by comprehensive sequencing analysis (11). Numerous immunoassays have been established for the serological characterization of *N. meningitidis* antigens (9). Capsular group identification can be conducted through PCR, AST, or WGS utilizing PorA and *fetA* typing data. The immunological detection of specific epitopes of PorA and PorB underpins the serological classification system that delineates the serotypes and serosubtypes of the species (9). Multiple-locus variable number tandem repeat analysis has been established as an alternative typing method for *N. meningitidis*. This technique indexes variability in short tandem repeated sequences to generate DNA fingerprints for epidemiological studies, demonstrating superior discriminatory power compared to MLST. Molecular procedures for genotyping are increasingly replacing phenotyping methods, such as serotyping (102). Pulsed field gel electrophoresis (PFGE), porA/porB sequencing, and MLST are among the available methods (45).

### 7.7 Metagenomic next-generation sequencing (mNGS) and real-time metagenomics (RTM)

Metagenomic next-generation sequencing (mNGS) is an advanced sequencing technology that facilitates the rapid analysis of nucleic acids in samples, including human and pathogenic microorganisms. This method allows for the comparison of these nucleic acids with human genome sequences and pathogenic microbial genome sequences, thereby determining the species and relative proportions of microorganisms present in the sample (103). The potential clinical applications of mNGS encompass novel pathogen detection, subtyping, outbreak investigation, identification of resistance mutations, and identification of organisms that are hard to culture (104). In recent years, mNGS has become an important diagnostic tool for sepsis and central nervous system infections, including bacterial meningitis (105). A recent meta-analysis assessed the efficacy of mNGS in diagnosing bacterial CNS infections from CSF, revealing a pooled sensitivity of 73.8% (95% CI: 59%–84%) and a specificity of 93.3% (95% CI: 87%–96%) (105). In CNS infections, especially in patients who have already started empirical antimicrobial therapy, mNGS offers significant advantages over conventional methods, such as bacterial culture, antigen tests, and PCR, in terms of its ability to detect pathogens (106). mNGS also has high diagnostic sensitivity in clinical samples other than CSF. In patients with clinical sepsis, the mNGS of plasma samples showed 94% sensitivity compared to blood cultures and identified a potential pathogen in 15% of the clinically negative samples (107). Although there are no comprehensive studies on the diagnostic sensitivity of mNGS specifically for *N. meningitidis*, it has been shown in several studies that mNGS is more successful than conventional methods in both CSF and non-CSF samples for the identification of bacterial meningitis agents, including *N. meningitidis*.

The combination of WGS and long-read sequencing techniques, such as nanopore sequencing, enhances the ability of researchers to address sequence variations in the numerous repetitive elements of *N. meningitidis*, thereby providing deeper insights into its evolution and epidemiology (96). Morsli et al. (108) demonstrate the feasibility of implementing RTM in a POC laboratory for single-instance diagnostics and genomic surveillance of pathogens associated with life-threatening meningitis. Due to its simplicity, speed, and the supplementary information obtained, RTM serves as an effective diagnostic tool for analyzing a prospective series of CSF samples from patients with meningitis (108). mNGS effectively identified *N. meningitidis* in the CSF of four patients with suspected meningococcal meningitis, and the genome of *N. meningitidis* was assembled for subsequent research (109). The pipeline analysis of antibiotic resistance and bacterial genotyping was conducted in-silico concurrently with sequencing, differing from traditional diagnostic methods. Furthermore, uncultured bacteria were effectively profiled in silico through pathogen genome analysis, irrespective of genome coverage. The RTM diagnosis was conducted within a total workflow duration of no more than four hours. Future advancements may involve the utilization of RTM in instances of undocumented meningitis and RNA virus cases to enhance the identification of causative pathogens in CNS diseases that are not routinely diagnosed (108, 109).

Bogaerts et al. (110) assessed the reduction of overall run time through shorter read lengths and the real-time analysis of isolate WGS data from Illumina instruments during sequencing. This approach allows for the integration of partially generated datasets into bioinformatics workflows for the typing and characterization of pathogenic isolates, which is critical when timely responses are necessary. An exhaustive performance evaluation was conducted at decreasing read lengths and coverages relative to sequencing run time. This study offers specific recommendations to ensure a minimum required level of performance, based on a previously described validation strategy. High-quality reference datasets for three pathogens, including *N. meningitidis*, were utilized, and multiple bioinformatics assays of public health interest were evaluated, which can also be adapted for other time-critical WGS applications (110).

Despite its transformative potential in diagnosing infectious diseases, mNGS has several disadvantages. One major issue is the lack of an agreed-upon standard for the number of readers to identify a pathogen in a sample. This uncertainty complicates the interpretation of mNGS results, as the vast amount of data generated can lead to false positives or negatives, especially if the read count is low or if there is environmental contamination. Additionally, there is a lack of knowledge regarding the actual limits of detection of LoD for each sequencing platform and protocol. Furthermore, the high cost and complexity of mNGS limits its widespread use (105, 111, 112).

### 7.8 Genome-wide association studies (GWAS)

Genome-wide association studies (GWAS) utilizing binary or single phenotype data have effectively identified genotypes associated with diseases and determinants of antimicrobial resistance (113). Earle et al. (114) conducted a bacterial genome-wide association study on *N. meningitidis*. A preliminary GWAS identified bacterial genetic variants, including single nucleotide polymorphisms (SNPs), linked IMD vs. carriage across multiple loci in the meningococcal genome, which encode antigens and other extracellular components, thereby affirming the polygenic characteristic of the invasive phenotype. The results were validated in an independent population of meningococci, revealing that DNA variants regulating fHbp production by the bacteria are significant, with elevated fHbp expression linked to invasion (114). The researchers utilized the variation in meningococcal genotypes, antigen types, and invasive potential to examine the meningococcal invasive phenotype through a hypothesis-free methodology, employing two GWAS of isolates from IMD and asymptomatic carriers (114). Maynard-Smith et al. (115) conducted an evaluation of serogroup Y meningococci in older adults. GWAS identified nonsynonymous polymorphisms primarily in the gene encoding transferrin binding protein B (tbpB) and, to a lesser extent, in the gene encoding MafI, an immunity protein belonging to the multiple adhesin family. A GWAS analysis identified nonsynonymous nucleotide polymorphisms in the tbpB gene that varied between younger and older age groups. Iron acquisition serves as a critical virulence mechanism, with variations in iron scavenging among meningococcal variants potentially leading to differences in colonization of individuals, influenced by factors such as age, nasopharyngeal microbiome, and nutritional status. This may result in age-related variations in the probability of colonized individuals developing IMD, offering a credible explanation for the recognized tendency of Y:cc23 meningococci to induce illness in older adults (115).

### 7.9 Culture-free WGS, selective whole genome amplification (SWGA)

WGS will increasingly be utilized in the diagnosis of infectious diseases; however, this will likely involve the advancement of non-culture-based techniques that directly sequence pathogen genomes from clinical samples (47). To enhance the representativeness of sequencing data in the absence of bacterial isolates, the advancement of culture-independent sequencing techniques is essential (47). To improve the detection and molecular characterization of meningococcal strains from clinical specimens, selective whole genome amplification (SWGA), a method based on isothermal multiple-displacement amplification, was validated (17, 47). SWGA is a culture-independent, targeted DNA enrichment technique that has been effectively validated on clinical specimens positive for meningococci. Subsequent to DNA extraction, denaturation, and neutralization, multiple-displacement amplification was conducted utilizing various components, including SWGA primers and phi29 polymerase or its thermostable variant EquiPhi20. The SWGA primers exhibit a binding affinity for meningococcal genomes, demonstrating nearly two orders of magnitude more coverage of *N. meningitidis* DNA in comparison to human DNA. The output consists of enhanced meningococcal DNA specimens and a tenfold increase in the quantity of clinical specimens that can be effectively sequenced by WGS. The overall average success rate of complete molecular typing exceeds 50%, with an increased number of genomes per μl correlating with elevated success rates. The incorporation of SWGA into genomic surveillance processes may enhance data quality and representativeness in the absence of cultures, hence bolstering public health surveillance and outbreak investigations (17). Itsko et al. (116) used a novel, culture-independent WGS methodology on nearly 400 clinical specimens obtained from meningococcal meningitis cases across six African nations (Burkina Faso, Ghana, Chad, Nigeria, Niger, and Togo) between 2017 and 2019. Approximately 50% of the specimens yielded high-quality whole-genome sequence data for thorough molecular profiling of the meningococcal pathogen. SWGA enrichment augmented the total sequence reads for meningococcal strains and yielded adequate high-quality molecular data for comprehensive strain characterization, encompassing MLST analysis and precise typing. Consequently, SWGA can serve as a viable option for genomic surveillance in instances with low bacterial culture rates. Although SWGA-based WGS can produce high-quality data for molecular typing, the serogroup identification rate in these specimens was merely 52% due to inadequate coverage of the capsule locus by SWGA heptamers and the stringent requirement for the integrity of all capsule genes by the computational pipeline. In conclusion, SWGA-based WGS methodologies yield insights into the genetic relatedness of circulating meningococcal strains and specific genes of interest. Incorporating this technique into genomic surveillance operations can enhance data quality and representativeness. This is particularly crucial in circumstances when acquiring bacterial isolates from meningococcal infections proves difficult (116).

### 7.10 Multiple cross-displacement amplification combined with real-time fluorescence

Multiple cross-displacement amplification (MCDA) isothermal amplification methods are promising. Due to its consistent temperature, MCDA reactions can be used in more conditions without thermal cyclers. Colorimetric changes or nanoparticle-based lateral flow biosensors (LFBs) enable easy diagnosis (117). Huang et al. (117) developed NM-RT-MCDA, a fast, sensitive, and specific *N. meningitidis* assay. This assay combines MCDA efficiency with real-time fluorescence detection precision. A one-hour turnaround time, including DNA extraction and amplification, makes NM-RT-MCDA a promising diagnostic tool for diverse clinical circumstances, potentially enabling timely IMD detection and intervention (117).

### 7.11 Genetic toolbox

The ease of mutant engineering has led the *Neisseria* community to neglect the development of more sophisticated tools for gene editing, particularly for *N. meningitidis*. Wuckelt et al. (118) highlighted that the efficient natural transformation of *N. meningitidis* allows for the rapid construction of bacterial mutants in which the genes of interest are interrupted or replaced by antibiotic-resistance cassettes. Although it facilitates the genetic characterization of this important human pathogen, it has limited the development of strategies for constructing marker less mutants without antibiotic-resistance markers. In addition, efficient tools for complementation or labeling are lacking in *N. meningitidis*. Wuckelt et al. (118) expanded the meningococcal genetic toolbox by developing new and efficient tools for the construction of marker less mutants (using a dual counterselection strategy), genetic complementation (using integrative vectors), and cell labeling (using a self-labeling protein tag) (118).

### 7.12 Pangenome graphs

Pangenome graphs offer an effective framework for representing and evaluating multiple genomes and their variants as a graph structure encompassing all forms of variation (95). The pangenome graph facilitates the concurrent discovery of structural variants, rearrangements, and minor variants, such as single nucleotide polymorphisms and insertions/deletions. Utilizing a pangenome graph rather than a singular linear reference genome enhances mapping rates and variation identification for both simulated and actual datasets of the pathogen *N. meningitidis*. Pangenome graphs present a promising methodology for comparative genomics and thorough investigation of genetic diversity in infectious illnesses. This new process, utilizing pangenome graphs, integrates variant analysis, genome assembly, population genetics, and evolutionary biology, therefore enhancing the scope of genomic comprehension and applications (95).

### 7.13 Machine learning

Using complete genomes as input to machine learning models allows quick strain classification using numerous genetic components. With SentencePiece or k-mer tokenization, Podda et al. (119) encoded complete bacterial genomes as a “bag-of-words” and evaluated them with machine learning. Instead of being limited by gene existence or short sequence reads, this approach would allow quick categorization using all information from various, presumably distant, genetic elements and intergenic regions. They subsequently classified *N. meningitidis* genomes' capsule B group genotype with 99.6% accuracy and the multifactor invasive phenotype with 90.2% accuracy in an independent test set. The ML technique performed well for both genetic (capsule group B assignment) and immunological (disease phenotype) classifications. This approach could be used for strain characterization, population genetics, antibiotic resistance monitoring, and epidemic investigation. Machine learning will soon provide statistical methods for fine mapping genome-wide association studies (GWAS) and expression quantitative trait loci (eQTL) data, predicting the disease impact of non-coding variants, and predicting sequence regulatory activity based on cross-species data (119).

## 8 Laboratory tests for meningococcal vaccine studies

The assessment of meningococcal vaccine effectiveness is challenging due to the low incidence of the disease; consequently, protection is estimated through serum bactericidal antibody (SBA) assays. Initial experiments on natural immunity determined a titer of ≥4 as the protective correlation for SBA assays utilizing human complement (hSBA); however, obtaining and standardizing human complement presents challenges (120). The SBA assay quantifies functional (bactericidal) antibody titers targeting capsular polysaccharides associated with meningococcal reference strains of groups A, C, W, or Y. The assay employs baby rabbit serum as an external complement source. An rSBA titer of ≥8 serves as the internationally accepted surrogate marker for protection against group C strains (46). The application of baby rabbit complements (rSBA assays), according to established guidelines for serogroups A and C, typically yields higher titers. Post-licensure effectiveness data for serogroup C conjugate vaccines indicate that rSBA titers ≥8 can be accepted as the correlate of protection for this serogroup; however, no formal thresholds have been established for serogroups A, W, and Y (46, 120). The MenB hSBA assay quantifies functional (bactericidal) antibody titers targeting sub-capsular protein antigens present in a selection of serogroup B meningococcal reference strains. This assay employs an external human complement. An hSBA titer of ≥4 serves as the internationally accepted surrogate marker for protection against group B strains (46). In 2022, Katz et al. (121) demonstrated the appropriateness of serogroup X SBA in clinical trials for prospective serogroup X polysaccharide-containing vaccines.

The antigens associated with MenB vaccines demonstrate significant variability in expression across strains, and evaluating immunogenicity following vaccination through conventional methods, such as the serum bactericidal antibody assay with human complement (hSBA), presents certain limitations (122). Various conservative bioinformatic methods have been developed to predict potential vaccine protection against MenB strains. These include the meningococcal antigen typing system (MATS), genetic MATS (gMATS), meningococcal antigen surface expression (MEASURE), and meningococcal deduced vaccine antigen reactivity (MenDeVAR) (122–124). The MATS method assesses the immunogenicity and cross-reactivity of fHbp, NadA, and NHBA peptides using three ELISA assays and a PCR for PorA (124). MATS assesses MenB strain coverage without the need for sera, facilitating the testing of extensive panels of bacterial isolates (124). The application of MATS in post-vaccine implementation surveillance may yield data on vaccine effectiveness in real-world settings and the duration of protection globally, which could inform the formulation of vaccine booster schedules, if required (122–124). gMATS is a predictive tool that correlates MATS results with antigen genotyping to assess strain coverage. This approach involves gMATS analyzing MATS results for fHBP and NHBA peptides, categorizing them as either coverage or non-coverage, while also incorporating the exact match of PCR for PorA VR2 (124). The meningococcal antigen surface expression (MEASURE) assay is a flow cytometric method that employs a fHbp-specific monoclonal antibody to quantify *in vitro* fHbp expression in fixed meningococcal cells (125). The flow cytometric MEASURE assay quantifies fHbp expression *in vitro*. Previous studies indicate that strains exhibiting a mean fluorescence intensity (MFI) greater than 1,000 are likely to be susceptible to killing by MenB-fHbp-induced antibodies. The MEASURE assay offers a more objective quantification of fHbp expression compared to the MATS assay, which relies on ELISA plates coated with antibodies specific to certain 4CMenB antigenic variants, thus allowing for independence from specific vaccine variants. The incorporation of the upstream intergenic sequence of the fHbp gene enhanced prediction accuracy by differentiating between low- and high-expressing strains (125).

The Meningococcal Deduced Vaccine Antigen Reactivity (MenDeVAR) Index, created by the University of Oxford, employs published strain characterization data to forecast vaccine coverage (125, 126). MenDeVAR is integrated within the PubMLST.org Neisseria database and automatically forecasts the strain coverage of both licensed fHbp-containing vaccines, utilizing the antigenic profiles of isolates submitted to the database. The MenDeVAR index classifies the isolates into four groups concerning MenB vaccines. Isolates that contain one or more specific antigenic variants included in MenB vaccines are classified as a “exact match.” Isolates with one or more antigenic variants demonstrating cross-reactivity in experimental studies are classified within the “cross-reactive” group. Isolates lacking sufficient data on their antigenic variants are categorized as “insufficient data,” while those containing only antigenic variants that did not demonstrate cross-reactivity in experimental studies are classified as “none.” The prediction was automatically calculated for all strains based on the fHbp peptide ID of each strain, in accordance with the established criteria (100, 125).

## 9 Safety considerations when handling meningococci in the laboratory

*N. meningitidis* is categorized as a hazard group 2 organism and must be managed under containment Biosafety Level 2 (BSL-2) or above. Laboratory personnel face risks when managing live meningococcal cultures and clinical samples with viable meningococci if proper safety measures are not upheld. Personnel must receive information and training regarding the biological hazards associated with meningococci and the signs and symptoms of IMD. It is essential that all standard operating procedures and risk assessments are executed and adhered to (44). The primary safety risk involves exposure to aerosols that contain viable meningococci. It is essential that any procedure capable of producing aerosols (e.g., handling liquid suspensions) is conducted with suitable containment measures, and, where feasible, the risk of aerosol generation should be eliminated from the process (44). To mitigate risks to medical laboratory scientists, the utilization of suitable personal protective equipment and biosafety cabinets is essential when handling organisms (127). Vaccination of all laboratory staff with available meningococcal vaccines is strongly recommended in appropriate immunization schemes (127). Booster doses of MenACWY-conjugated vaccines have been recommended every five years for laboratory personnel routinely exposed to isolates of *N. meningitidis*. A single dose of MenB vaccines has been recommended at one year after completion of primary vaccination and every 2–3 years thereafter (128, 129).

## 10 Discussion

Patients suspected of having IMD require immediate treatment, irrespective of laboratory confirmation (1, 11). All suspected, probable, and confirmed cases of meningococcal disease must be reported immediately to the relevant health department to facilitate the implementation of appropriate prevention and control measures (7). High-quality epidemiological surveillance data and the collection of invasive isolates from a diverse and representative population are essential for informing prevention and control strategies for IMD (11, 17, 129). These data serve to monitor disease trends, calculate disease burden, identify causative serogroups and antibiotic resistance, detect new cases and outbreaks, characterize risk factors and severe outcomes, guide immunization strategies, assess vaccine impact, and inform the development of new vaccines (11). The presence, type, and effective use of surveillance systems vary considerably between countries and regions. Various factors, such as countries' economic conditions, health policies, laboratory facilities, and geographical limitations, influence the choice of a surveillance system (131). To reduce underdiagnosis, it is necessary to provide a well-established surveillance system, improve diagnostic methods, increase the awareness of clinicians, and ensure that patients receive a timely and accurate diagnosis and notification of diseases (131). Laboratory confirmation and characterization of *N. meningitidis* are essential. The first step in the diagnosis of IMD is clinical suspicion. The main difficulty in diagnosing IMD is considering common infections rather than meningococci due to nonspecific symptoms at the onset of the disease. Atypical presentation (symptoms such as gastrointestinal symptoms), atypical clinical courses (septic arthritis, pneumonia, pericarditis, and urethritis), and lifelong disease potential, including the elderly, should be kept in mind (1, 12).

At present, no biomarker or combination of biomarkers demonstrates adequate diagnostic accuracy to serve as rule-in or rule-out tests for IMD (87). For microbial detection of *N. meningitidis*, microbiological tests, point-of-care assays, molecular assays, and next-generation sequencing techniques have been used. All these diagnostic methods have their own pros and cons (Table 1). Microbiological tests include bacterial culture, Gram staining, isolate preservation, and DNA extraction. The advantages of microbiological tests are that they are rapid and cheap, and they allow isolates to be archived for future analysis. However, prior antimicrobial treatment might be related to negative results. Point-of-care tests (latex agglutination tests and lateral flow assays) are also cheap and easy to perform, reporting results rapidly; however, results can be subjective and sensitive to prior antibiotic treatment. Molecular assays (e.g., rt-PCR, LAMP assay) are sensitive and specific and mainly designed for nucleic acids; therefore, they are not reliant on the presence of viable bacteria. However, the cost of these is higher than others, requiring equipment and personnel with expertise. Comprehensive and open-access meningococcal classification data, which generally rely on continually evolving immunologic or molecular methods, are crucial for global meningococcal surveillance. The surveillance network for IMD in Europe started in 1999 as a part of the Invasive Bacterial Infections Surveillance project (132). The network aims to enhance epidemiological data on IMD in Europe, characterize meningococcal isolates, and promote international collaborations. Numerous reference laboratories across Europe utilize the European Meningococcal Epidemiology in Real Time (EMERT) database, which has been collecting real-time data throughout Europe since January 2007 (http://emgm.eu/emert/). The European Surveillance System (TESSy) will incorporate molecular typing data for meningococcal disease (70). WGS data are frequently annotated and deposited in the meningococcal PubMLST database. The PubMLST website provides comprehensive phenotypic, genotypic, and epidemiological data (133). MLST data are available online at http://pubmlst.org/neisseria/, where each sequence is assigned an allele number based on its sequence after querying the MLST profile database. The PubMLST database contains 78,385 *N. meningitidis* isolates and 40,011 genomes, with the last access recorded on 7 February 2025. Another freely accessible website offering high-quality and updated genomics is MicroScope: Microbial Genome Annotation & Analysis Platform (13, 134). Next-generation sequencing tests (Illumina sequencing, metagenomic analysis, genome analysis, nanopore) allow the bacterium to be comprehensively characterized; however, the high cost and requirement of expertise and costly laboratory infrastructure are disadvantages. The WHO has recently endorsed a global initiative to eliminate meningitis worldwide by 2030. Diagnosis and treatment constitute one of the five pillars of the roadmap for this global vision, emphasizing the necessity for comprehensive and cost-effective diagnostic methods to improve surveillance.

**Table 1:** Features of the methods used for laboratory diagnosis of invasive meningococcal disease (9, 44, 47, 61, 66, 74, 75, 87, 88).

Method	Advantages	Disadvantages	Sensitivity, %	Specificity, %
Gram Staining	• Rapid (in minutes) • Inexpensive, accessible, easy • Not affected by antibiotic usage	• Low sensitivity with low bacterial load • Low sensitivity in case of low amount of CSF not allowing centrifugation	66.0–97.2	97.4–100
Culture	• Gold standard, definite identification • Allows for antibiotic susceptibility test • Isolates can be archived, allows for further characterization • Guiding the determination of vaccine content	• Time-consuming (in days) • Difficulty in storage and transport of the samples • Results are affected by antibiotic usage	55–63	100
rt-PCR	• Rapid (in hours) • High sensitivity and specificity • Not affected by antibiotic usage	• Expensive, requires special equipment	89.5–96	94.5–100
MALDI-TOF	• Rapid (in minutes) • Rapid identification from positive blood culture bottle or CSF samples	• Requires approximately 10^4^–10^6^ CFU/ml bacteria • Needs to be updated in the identification of *N. meningitidis*	100%	52–92
Antigen tests	• Rapid (in minutes) • Requires very little CSF sample • Easy to apply • Easy storage of test kits	• Absence of serogroup B in the panel • The sensitivity range being wide (or variable)	32.0–95.0	90.0–93.8
qPCR-HRM	• Less expensive than the RT-PCR • Probe-free method		-	-
LAMP	• Easy, fast, and inexpensive • CSF, blood, naso/oropharyngeal swab can be used		84–100	94–100
			ctrA 89/100	*ctrA* 100/98.9
			*NMO_1242* 100	*NMO_1242* 100
WGS	• Extensive characterization of the isolate	• Requires expensive laboratory infrastructure and technical knowledge • Still useful in epidemiology rather than diagnosis		

CSF, cerebrospinal fluid; rt-PCR, real time polymerase chain reaction; MALDI-TOF, matrix assisted laser desorption ionization time of flight mass spectrometry; qPCR-HRM, qualitative PCR with high-resolution melting; LAMP, loop-mediated isothermal amplification; WGS, whole genome sequencing.

Some features of *N. meningitidis* may also cause difficulties in laboratory diagnosis. *N. meningitidis* is a fastidious bacterium that dies within hours on inanimate surfaces (1, 13, 116). It is challenging to grow clinical samples in culture if they cannot reach the laboratory quickly and under suitable conditions. The genetic diversity of *N. meningitidis* and the high degree of genetic relatedness with commensal *Neisseria* species can make molecular tests difficult to detect. Sirluck-Schroeder et al. (64) suggested that some commercial tests target only encapsulated strains, and that these strains do not represent all strains capable of causing IMD. Using non-capsular genes, such as *sodC, MetA, and tauE*, may also detect meningococci in *ctrA*-negative samples and reduce false-negative results (64, 72). The severity of IMD has led to the extensive use of genomic technologies for a detailed molecular epidemiological investigation of *N. meningitidis*. This has disclosed a restricted variety of hypervirulent lineages that typically segregate into distinct geographical areas. The organism's capacity for horizontal gene transfer allows individual clones to exhibit significant variation in antigen expression, such as capsular polysaccharides. Emerging clones have the potential to trigger significant epidemics and disseminate swiftly both domestically and globally (93). The abundance of human DNA in the environment may pose challenges when sequencing the genome for meningococcus from clinical samples. Therefore, researchers are developing methods to enrich the desired bacterial DNA. Itsko et al. (116) reported that they obtained good results with the SWGA method they developed to detect meningococcal DNA from clinical samples with a low bacterial load. They suggested that it is a relatively inexpensive method and will contribute to enhancing molecular surveillance in regions with a low culture rate (116).

New molecular assays for identifying invasive meningococcal infections are now being developed; nevertheless, they remain mostly unavailable, particularly in resource-limited settings. Consequently, the primary objective is to precisely diagnose the index patient and swiftly isolate and administer treatment. Patients with clinical symptoms should therefore be tested using PCR, Gram staining, and culture. RT-PCR-based or semiquantitative assays, albeit being comparatively more costly, are helpful in the rapid identification and isolation of the index case and the chemoprophylaxis of contacts. The approach will provide prompt notification and registration of patients to the health authority. LAMP will be especially beneficial during periods of elevated disease activity in the field. WGS also provides us more information about N. meningitidis at the molecular level. WGS remains a costly test and is not a suitable choice for initial diagnostic and therapy strategies in numerous countries worldwide. The extensive application of WGS in reference laboratories will facilitate molecular-level epidemiology of infectious diseases, epidemic identification, assessment of vaccine coverage rates, and the creation of novel vaccines. Despite significant advancements in the identification of meningococcal infections, disparities in access and implementation persist across different regions. The identification, notification, diagnosis, and treatment of patients with IMD, along with the global harmonization of immunization regimens, would significantly enhance disease control.

In conclusion, the diversity of *N. meningitidis* necessitates precise identification of species and capsules for effective diagnosis, treatment, surveillance, and public health interventions. This guarantees the implementation of pertinent measures for case and outbreak management and informs subsequent vaccine development. IMD is an important and emergent problem due to its high mortality rate and potential permanent sequelae. With appropriate treatment, the early detection of IMD cases at high risk of death or serious illness will contribute to improved survival and reduced sequelae. Therefore, methods that will provide an early diagnosis of IMD have significant clinical value. Rapid diagnostic methods, such as rt-PCR, should be generalized. Laboratories in regions with limited resources should cooperate with regional or global reference laboratories to benefit from new test methods, such as WGS. Gram staining should not be neglected, as it is a simple, inexpensive, and easily accessible test and has an important place in diagnosis. Clinicians should be vigilant for atypical IMD presentations and should be alert to the possibility of infections with new serogroups, even if vaccination is successful. Healthcare professionals should be provided with up-to-date information on diagnosis and treatment. Currently available, demonstrably effective vaccines against IMD include polysaccharide vaccines for serogroups A, C, W, and Y, and subcapsular protein vaccines for serogroup B (19, 20, 135–139). The recently licensed MenABCWY vaccine may crucially support broad expansion of meningococcal vaccination programs by enabling comprehensive protection against all five major disease-causing serogroups using a single vaccine and vaccination schedule, along with fewer injections (140, 141). Positive clinical data for the WHO-prequalified MenACWYX vaccine encourage its replacement of MenA-TT within the African meningitis belt to expand the MenA-TT program's success to currently circulating serogroups, including MenX (142). The implementation of available meningococcal vaccines in immunization programs is a crucial step in the prevention of IMD. In addition to playing a key role in the fight against antimicrobial, expanding meningococcal vaccination programs to include vaccination against all five major serogroups and in all risk-based age groups thus represents a key strategy in pursuit of the WHO's goal of defeating meningitis by the year 2030. A key pillar of the WHO roadmap for defeating IMD by 2030 is prevention and epidemic control, which can largely be achieved using effective prophylactic vaccines (143). Importantly, the roadmap aims to reduce vaccine-preventable meningitis cases, including those caused by *N meningitidis*, by 50%, and deaths by 70%, compared with 2015 estimates across global regions; thus, all countries are urged to promote increased meningococcal vaccination. Microbiology reference laboratories play a vital role in the public health system, particularly during pandemics (144). Microbiology reference laboratories significantly influence public health at regional, national, and international levels, necessitating ongoing support for their operations.
